# Targeted spleen modulation: a novel strategy for next-generation disease immunotherapy

**DOI:** 10.7150/thno.111116

**Published:** 2025-03-18

**Authors:** Wei Dong, Yucheng Li, Qiaoman Fei, Senyang Li, Xinrui He, Yichao Chai, Junyi Zhou, Yujin Zong, Jing Geng, Zongfang Li

**Affiliations:** 1 National and Local Joint Engineering Research Center of Biodiagnostics and Biotherapy, The Second Affiliated Hospital, Xi'an Jiaotong University, Xi'an, China.; 2 Department of Geriatric General Surgery, The Second Affiliated Hospital, Xi'an Jiaotong University, Xi'an, China.; 3 Center for Tumor and Immunology, The Precision Medical Institute, Xi'an Jiaotong University, Xi'an, China.; 4 Shaanxi Provincial Clinical Medical Research Center for Liver and Spleen Diseases, CHESS-Shaanxi consortium, The Second Affiliated Hospital, Xi'an Jiaotong University, Xi'an, China.; 5 Key Laboratory of Biomedical Information Engineering of Ministry of Education, Department of Biomedical Engineering, School of Life Science and Technology, Xi'an Jiaotong University, Xi'an, China.

**Keywords:** Spleen, Splenic neuro-immune interplay altering immunocompetence, Physical stimulation immunomodulating splenic immunity, Cholinergic anti-inflammatory pathway.

## Abstract

The spleen, the largest lymphatic organ, comprises a diverse array of immunocytes in approximately one quarter of the body, including T cells, B cells, natural killer cells, and myeloid cells (such as dendritic cells, neutrophils, myeloid-derived suppressor cells, and macrophages). These immune cells undergo dynamic transitions and mobilization, enabling the spleen to execute a wide range of immunological functions. The spleen's structural organization and multicellular composition, along with its reservoir of lymphocytes, facilitate the capture and clearance of blood-borne antigens while also orchestrating both innate and adaptive immune responses. Additionally, the spleen plays critical roles in hematopoiesis and the removal of aged or damaged red blood cells. Despite being innervated by sympathetic (catecholaminergic) nerve fibers, the spleen lacks parasympathetic (vagal or cholinergic) innervation. The neuroimmune axis, particularly the interplay between sympathetic and parasympathetic nervous system immune circuits, significantly influences disease onset and progression. Extensive research employing physical, genetic, and pharmacological approaches has sought to directly modulate splenic immunocytes and activate neuroimmune interactions to restore immune homeostasis and counteract disease. Two primary mechanisms underlie these immunomodulatory interventions: (1) the cholinergic anti-inflammatory pathway, wherein norepinephrine released by splenic catecholaminergic fibers binds to β2-adrenergic receptors on CD4⁺ T cells, triggering acetylcholine secretion, which in turn suppresses inflammatory cytokine production in macrophages via α7 nicotinic acetylcholine receptor signaling, and (2) direct immunomodulation of splenic immunocytes, which regulates key genes and signaling pathways, alters cytokine secretion, and modulates ion flux to influence cellular functions. Among various therapeutic strategies, physical methods, particularly electrical stimulation and splenic ultrasound stimulation, have demonstrated the greatest promise for clinical applications in splenic immunomodulation and disease management.

## 1. Introduction

The spleen, the largest secondary lymphoid organ, plays a crucial role in both innate and adaptive immunity, as well as in hematopoiesis and red blood cell (RBC) clearance 1, 2. It serves as a reservoir for a diverse array of immune cells, including T cells, B cells, natural killer (NK) cells, and myeloid cells such as dendritic cells (DCs), neutrophils (NPs), myeloid-derived suppressor cells (MDSCs), and macrophages (Mφs). Notably, splenic lymphocytes comprise approximately one-quarter of the body's total lymphocyte population, highlighting the organ's immunological significance 1, 2. The spleen's microanatomy, characterized by myeloid-rich red pulp and lymphoid-rich white pulp, provides a specialized environment for immune cell maturation, antigen presentation, and immune regulation. Through these functions, the spleen contributes to host defense against pathogens, tumor surveillance, and immune tolerance, while also playing a role in preventing excessive inflammation and autoimmunity 3.

Beyond its immunological functions, the spleen is increasingly recognized as an integral component of the neuroimmune axis. A dense network of sympathetic nerve fibers innervates the spleen, primarily modulating immune responses through catecholaminergic signaling 3. However, unlike many other lymphoid organs, the spleen lacks significant parasympathetic or direct vagal innervation 3, 4. Emerging research suggests that neural inputs influence splenic immune cell activity, impacting processes such as cytokine production, leukocyte trafficking, and inflammatory responses. These findings have sparked growing interest in the therapeutic potential of neuroimmune modulation as a means to regulate immune function and treat immune-related disorders.

In recent years, considerable effort has been devoted to exploring novel strategies for modulating splenic immune activity, particularly through physical interventions such as electrical and ultrasonic stimulation. Preclinical studies have demonstrated that targeted neuro-/immune modulation of the spleen can effectively influence immune cell dynamics, suppress excessive inflammation, and enhance protective immune responses. These findings pave the way for potential clinical applications in treating autoimmune diseases, inflammatory disorders, and infections.

Given these advances, a comprehensive evaluation of the spleen's architecture, immune cell dynamics, and neuroimmune interactions is essential for understanding its broader role in immune regulation. This review aims to synthesize current knowledge on splenic immunology and neuroimmune signaling while highlighting emerging therapeutic strategies. By integrating these perspectives, we provide insights into the spleen's potential as a target for novel immunotherapies and bioelectronic medicine, offering a foundation for future translational research and clinical applications.

## 2. Spleen, and its role in the occurrence and progression of various diseases

### 2.1 Overview of splenic architecture

**Figure 1:**
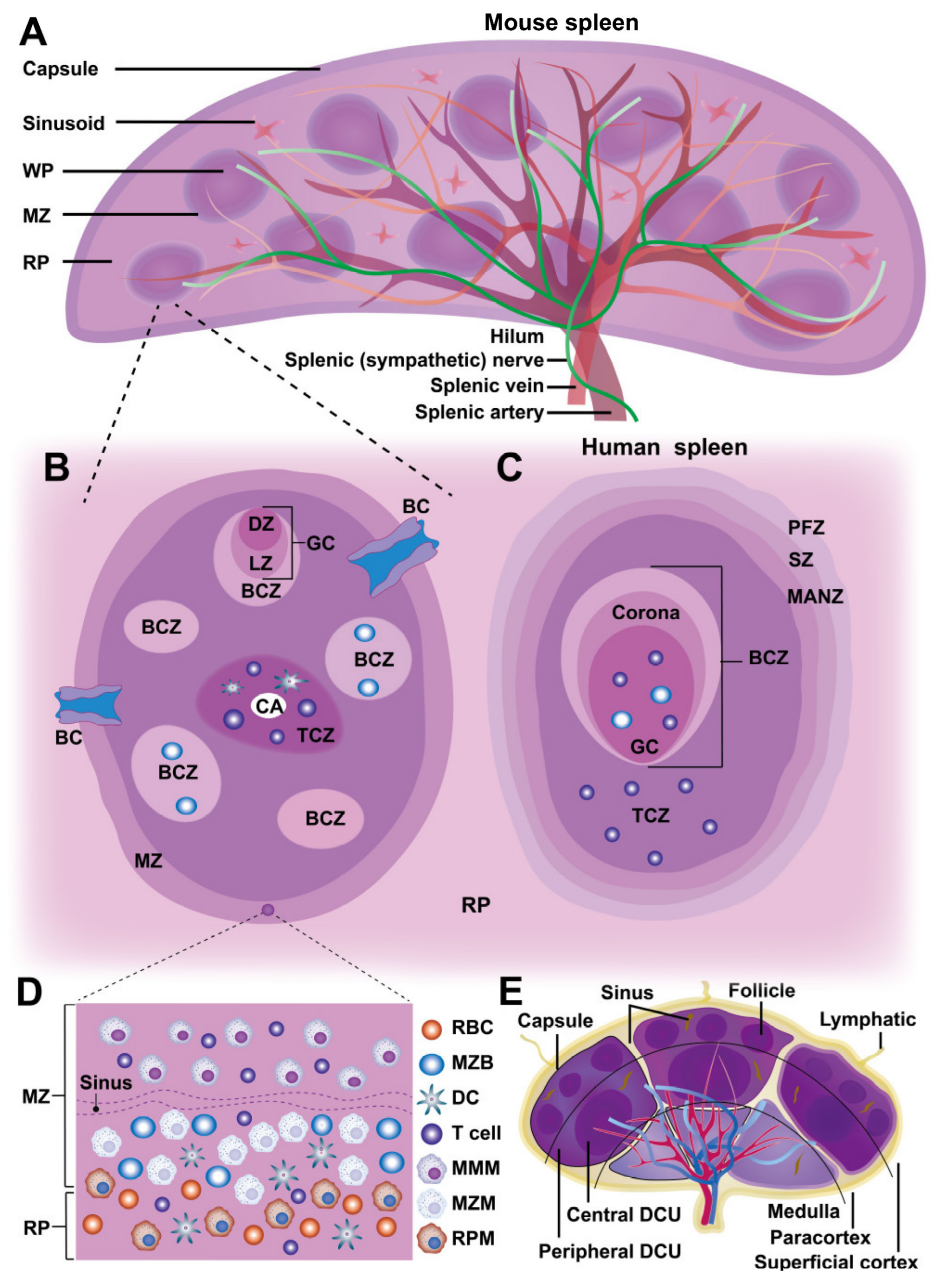
Schematic diagram of spleen architecture. **(A)** The panoramic structure of the mouse spleen. **(B-C)** Cross-sectional illustrations of a small portion of the white pulp (WP) in both mouse and human spleens, highlighting their structural differences. Notably, the organization of T cell zones (TCZ) and B cell zones (BCZ) within the WP is shown, with the light zone (LZ) and dark zone (DZ) clearly depicted. The border between the WP and red pulp (RP) is also shown, with the marginal zone (MZ) in mice and the perifollicular zone (PFZ) in humans. The PFZ includes the mantle zone (MANZ), superficial zone (SZ), and the outer layer of the PFZ. **(D)** The precise layering and composition of macrophage (Mφ) subsets in the MZ of the spleen, as known in mice. CD169^+^ marginal metallophilic Mφs (MMMs) form a concentric ring around the WP, along with MZ Mφs (MZMs) and MZ B cells (MZBs), but not for humans. In humans, MZB cells surround activated B cells, forming a germinal center (GC) and corona. The homeostatic location of dendritic cell (DC) subsets in mice is also depicted, with cDC2s in the bridging channel (BC), and cDC1s in the TCZ, MZ, and RP. The release of blood into the MZ from the central arteriole (CA) is shown. The mouse MZ is well-defined, with a BC that is absent in humans. In mouse infectious models, antigen-specific T lymphocytes move through the BC from the TCZ in the WP to the RP and ultimately enter the circulation via venous drainage. **(E)** Schematic representation of lymph node (LN) architecture. A midsagittal section of a LN is idealized to contain three lymphoid lobules. Each lobule is centered under its own afferent lymphatic vessel. The follicles and interfollicular cortex within the lobules constitute the superficial cortex, while the deep cortical units (DCU) form the paracortex, and the medullary cords and medullary sinuses make up the medulla. Arterioles (red) and venules (blue) are located within the medullary cords. Arterioles arborize in the paracortical cords of the peripheral DCU and interfollicular cortex, leading to capillary beds (purple). Capillaries drain into high endothelial venules, which then condense repeatedly in the interfollicular cortex and peripheral DCU before transitioning to medullary venules at the corticomedullary junction. Lymph from the afferent lymphatic vessel flows over the apical surface of the lobule in the subcapsular sinus, migrates through lateral transverse sinuses, and passes through medullary sinuses surrounding the medullary cords before exiting via the efferent lymphatic vessel in the hilus. B lymphocytes home to follicles in the superficial cortex, where they interact with follicular DCs, while T lymphocytes home to the DCU in the deep cortex (paracortex), where they interact with DCs. The DCU is organized into a center and a periphery, with the peripheral DCU and interfollicular cortex serving as transit corridors for arterioles, high endothelial venules, and paracortical sinuses.

Anatomically, the spleen comprises a capsule, trabeculae, and lymphatic tissues (Figure 1A). Its outer capsule consists of dense connective tissue with smooth muscle fibers, while trabeculae extend inward, dividing the parenchyma into compartments and supporting blood vessels. Internally, the spleen is divided into red pulp (RP) and white pulp (WP), separated by the marginal zone (MZ) in rodents or the perifollicular zone (PFZ) in humans (Figure 1B-D). The MZ in rodents is well-defined and facilitates antigen transport from the blood to immune cells, whereas the PFZ in humans is less distinct and lacks a comparable bridging channel (BC). This variation affects how efficiently antigens are presented to lymphocytes, potentially influencing immune response dynamics. Although the WP comprises less than a quarter of the spleen, it serves as the primary site for adaptive immune responses, while the RP, which makes up most of the spleen, functions in blood filtration and recycling. Unlike lymph nodes, the spleen lacks lymphatic vessels, relying entirely on blood circulation for cellular and antigen transport (Figure 1B, E).

#### 2.1.1 White pulp

The WP consists of small, spherical lymphatic follicles primarily composed of densely packed B lymphocytes surrounding the central artery (CA). In rodents, the CA is encircled by the marginal zone (MZ), which merges with the red pulp (RP) cell cords 5. However, in humans, this structure is replaced by the perifollicular zone (PFZ), which lacks a well-defined MZ or BCs. The WP also contains periarterial lymphatic sheaths (PALS), a network of diffuse lymphatic structures that extend to follicular edges and surround the CA. As the CA passes through large trabeculae before reaching the splenic parenchyma, its associated PALS plays a critical role in lymphocyte organization. In rodents, PALS is distinctly structured into two specialized immune zones: the T cell zone (TCZ) and the B cell zone (BCZ), whereas in humans, the organization appears more diffuse. The TCZ serves as the primary site for T cell activation, where T cells interact with antigen-presenting dendritic cells (DCs) to initiate cellular immunity. In contrast, the BCZ is where B cells undergo germinal center (GC) formation to facilitate humoral immune responses and antibody production 5. In murine spleens, the well-structured MZ plays a crucial role in antigen capture, efficiently directing macrophages and MZ B cells to process circulating antigens. In human spleens, however, antigen uptake and presentation are thought to rely more on DC migration from the PFZ to the WP, potentially leading to differences in immune surveillance and response to systemic infections. These structural variations suggest that antigen presentation and adaptive immune activation may follow distinct pathways between species, highlighting the importance of considering species-specific differences when extrapolating murine immunology findings to human disease models. Furthermore, research has shown that chemokine-mediated immune cell localization in the WP differs between species. For example, CCR7 and its ligands (CCL19, CCL21) are essential for guiding T cells to the TCZ, whereas CXCL13 plays a key role in recruiting B cells to the BCZ, where its receptor, CXCR5, is highly expressed 6, 7.

The spleen, a peripheral circulatory organ, contains LN-like structures in the WP but differs from LNs in key aspects (Figure 1E). Unlike LNs, the WP lacks a capsule separating it from the RP; instead, a cellular boundary of innate immune cells demarcates the WP, which is well-defined in mice but only partially in humans (Figure 1B, E). Despite this, antigens larger than 60 kDa cannot freely enter the WP but are transported by cells from the MZ 8. Additionally, the WP lacks lymphatic vessels, suggesting that it does not receive cells and antigens via lymphatic drainage. While often considered the draining secondary lymphoid organ (SLO) for the peritoneum in murine studies, most intraperitoneally injected antigens track to mediastinal LNs rather than the spleen 9. Thus, the WP primarily serves as the SLO of the circulatory system, akin to LNs in tissue antigen monitoring.

#### 2.1.2 Red pulp

The RP parenchyma comprises a branched reticular network housing lymphocytes, NPs, Mφs, and mast cells, organized into splenic cords that encircle large venous sinusoids. Its primary function is filtering aged, apoptotic, or opsonized cells while detecting pathogens and tissue damage 5. Blood enters the spleen through terminal arterioles and is released into an open circulatory system, lacking traditional endothelial linings. During this process, the RP filters and removes aged RBCs, with RP Mφs (RPMs) specifically phagocytosing deformed, infected, or dysfunctional RBCs. After percolating through the splenic cords, the blood is collected into the splenic sinusoids forming the venous sinusoidal system, and eventually returns to the circulatory system via the efferent vein. Thus, the RP plays a crucial role in immune cell phagocytosis, facilitated by the slow blood flow in this region.

Although adaptive immune responses to systemic antigens are initiated in the WP, immune effector functions often occur within the RP. In addition to Mφs, the RP also contains other immune cells, including T cells, DCs, and NK cells, all of which contribute to the innate immune response within the RP 10. Moreover, MDSCs dynamically change both in location and proportion during immune-inflammatory responses, thus enabling rapid reactions to insults and modulating the adaptive immune response in the RP. Plasmablasts also migrate from the WP to the RP in response to chemokine CXCL12, where they produce antibodies that circulate throughout the body 11. Effector CD8^+^ T cells also emigrate to the RP to combat antigen invasion 12. Additionally, the RP supports extramedullary hematopoiesis and serves as a reservoir for monocytes, platelets, and RBCs 13.

#### 2.1.3 Marginal zone

The MZ, a crucial interface between the WP and RP, exhibits notable structural and functional differences between rodents and humans. In rodents, the MZ consists of multiple cellular layers containing naive B cells, Mφs, NK cells, DCs, and circulating blood cells. A distinctive population of innate-like B cells, known as MZ B cells (MZBs) is anchored by integrins LFA-1 and α4β7, which bind to ICAM-1 and VCAM-1, respectively, along with chemotactic signals from sphingosine-1-phosphate (S1P) 14. Additionally, the murine MZ harbors two specialized Mφ subsets: marginal metallophilic Mφs (MMMs), expressing receptors such as MOMA1 and MARCO, and MZ Mφs (MZMs), which express sialoadhesin markers such as CD169 (Siglec-1) 15. These Mφs are integral to the antigen capture, processing, and presentation, activating B cells for IgM production and serving as antigen-presenting cells (APCs). MZ-resident Mφs also express pattern recognition receptors (PRRs) essential for clearing blood-borne pathogens. At this WP-RP interface, specialized leukocytes, including DCs and MZBs, capture and transport blood-borne antigens to the WP for surveillance by T and B cells 6. Disruption of the MZ significantly reduces T and NK cell activation, highlighting the cooperative role of MZMs and DCs in natural killer T (NKT) cell responses 16. Furthermore, lymphocytes and accessory cells from the WP are derived from the MZ, whose reticular cells express mucosal addressin cell adhesion molecule-1 (MAdCAM-1), an essential homing receptor that facilitates the entry of lymphocytes into both the MZ and WP 17.

BCs are defined by gaps in the ring of metallophilic Mφs and contain a specialized subset of CD4^+^ DCs, which are retained through oxysterol ligands produced by local stroma cells 18. These BCs can generate the chemokine CCL21, which recruits naive and activated lymphocytes, guiding their migration through the MZ to RP before reentering circulation 12. Some studies suggest that efferent lymphatics may originate in the WP, allowing a fraction of lymphocytes to exit via lymphatic vessels, though this hypothesis requires further validation 13.

### 2.2 Splenocytes-mediated immunity and its role in disease pathogenesis and therapy

To delineate the immune functions of the spleen in relation to disease pathogenesis and therapeutic interventions, we summarized the origins, activities, and dynamics of key splenocyte populations based on their roles: (1) splenocytes resident in the spleen prior to immune activation; (2) cells recruited in response to pathological states; (3) cells produced or amplified locally within the spleen; and (4) splenocytes migrating from the spleen to niduses.

#### 2.2.1 Resident lymphocytes and phagocytes

**Figure 2:**
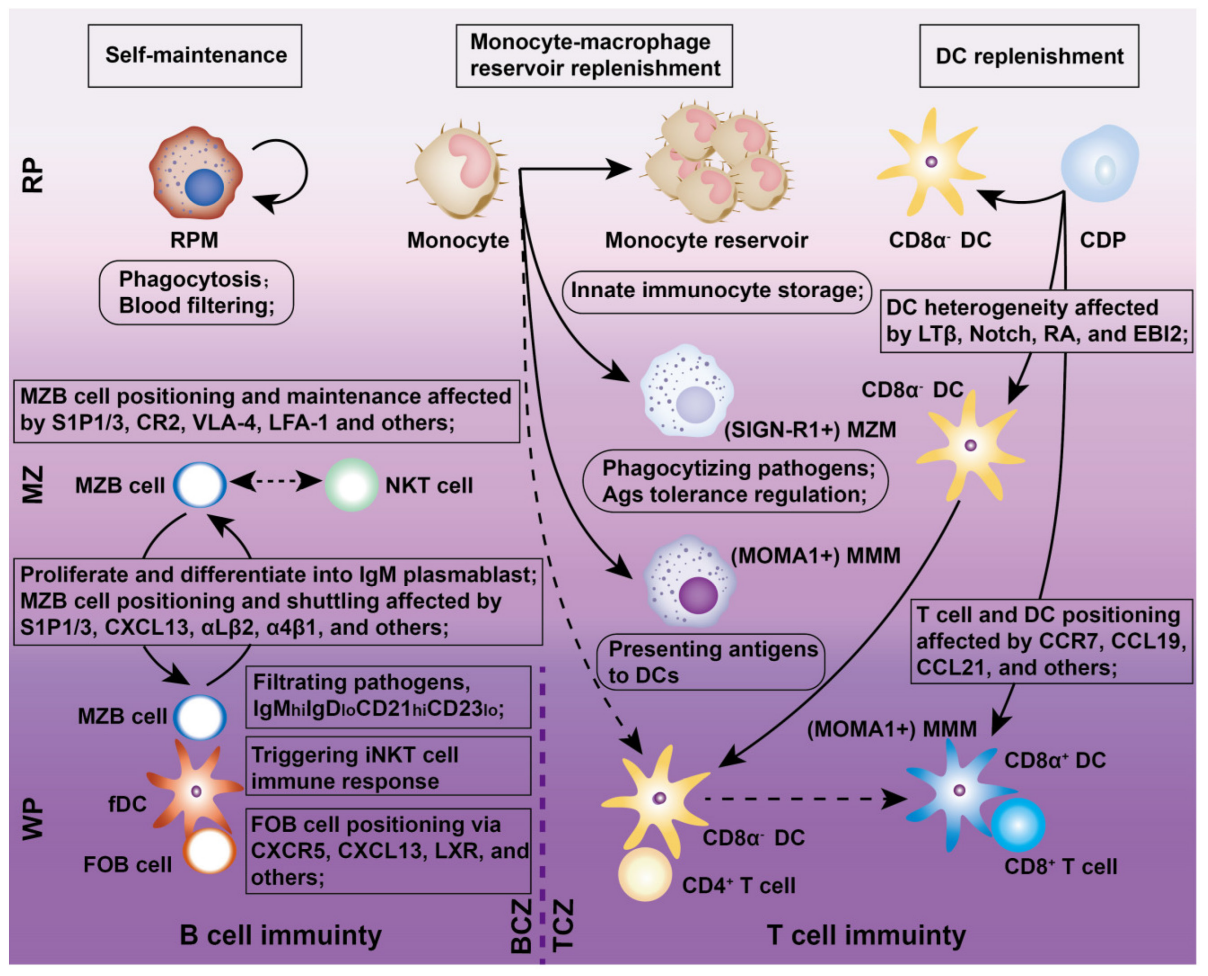
Schematic representation of splenic immune cells and their functions against pathogens. The illustration provides an overview of various innate and adaptive immunocytes within the spleen, highlighting their distinct roles in responding to pathogens. It also depicts cell localization, motility, and interactions within different splenic compartments. Abbreviations: Ag, antigen; CDP, common dendritic progenitor; CR2, cannabinoid receptor 2; fDC, follicular DC; FOB, follicular B cell; GRK2, guanine nucleotide-binding protein-coupled receptor kinase-2; LTβ, lymphotoxin beta; LXR, liver X receptor; RA, retinoic acid; S1P1, sphingosine-1 phosphate-1.

The spleen hosts all major mononuclear phagocytes (e.g., Mφs, DCs, and monocytes), which are responsible for pathogen recognition, immune regulation, apoptotic cell clearance, and disease modulation (Figure 2).

The spleen contains four distinct Mφ subtypes: MZMs, MMMs, RPMs, and tingible body Mφs, each residing in specific niches and expressing unique PRRs and scavenger receptors for specialized immune functions (Figure 2). MZMs and MMMs are derived from bone marrow (BM) progenitors and depend on macrophage colony-stimulating factor (M-CSF) and liver X receptor alpha (LXRα) for development. MZMs, characterized by the expression of MARCO and SIGN-R1, are localized within the MZ and interact with MZBs 19. In contrast, MMMs, which express SIGLEC1/CD169 and MHC II, extend cellular processes from the MZ into the WP, where they can present antigens to DCs 20. Both MZMs and MMMs share a common BM monocyte lineage and play critical roles in maintaining tolerance to self-antigens. During inflammation, these populations are rapidly replenished by BM-derived monocytes. RPMs, self-renewing and M-CSF-independent, originate from yolk sac progenitors and reside in the RP, where they filter blood, clear pathogens, apoptotic cells, and debris, and “groom” RBCs by removing abnormal inclusions (e.g., denatured hemoglobin). Inflammatory conditions may partially replenish RPMs with BM-derived monocytes 21. Tingible body Mφs reside in B cell follicles and are responsible for clearing apoptotic B cells generated during selection processes, such as affinity maturation and class switching, in the GC's light zone (LZ) 22.

**Figure 3:**
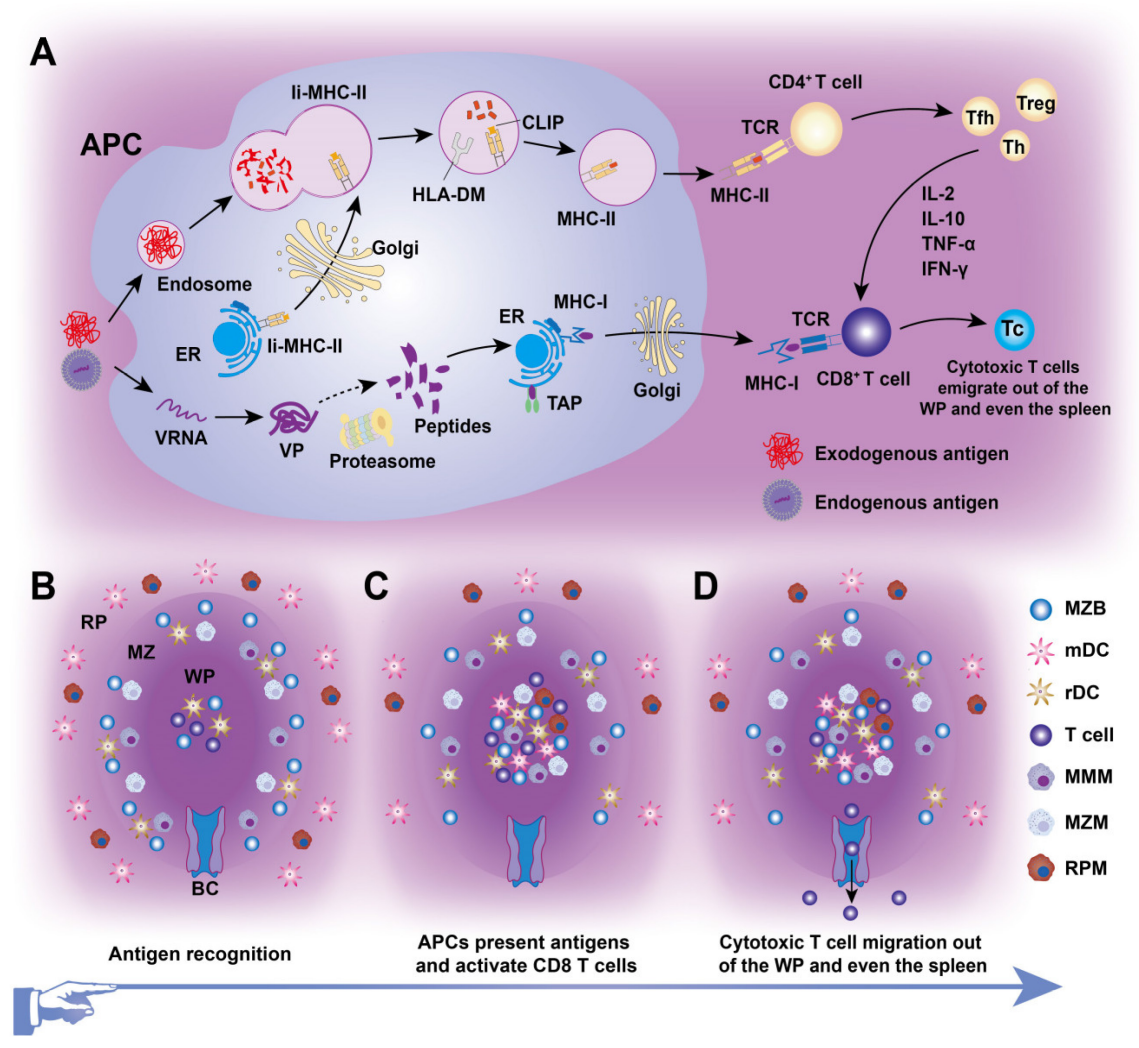
Diagram of cellular immunity mediated by splenic immunocytes. **(A)** Intracellular antigen presentation occurs via two primary routes: (i) MHC-I pathway: Intracellular antigens are hydrolyzed into peptides by proteasomes in the cytoplasm, then transported into the endoplasmic reticulum (ER) lumen via transporters associated with antigen presentation (TAP). There, peptides are loaded onto MHC-I molecules, and the resulting peptide-MHC-I complexes are transported through the Golgi apparatus to the antigen-presenting cell (APC) membrane. This process activates CD8^+^ T cells, which, under the influence of cytokines such as IL-2 and IL-10, differentiate into cytotoxic T cells, initiating cellular immunity. (ii) MHC-II pathway: MHC-II molecules are synthesized in the ER and form a complex with the invariant (Ii) chain, which prevents premature peptide binding. This Ii-MHC-II heterotrimer is transported through the Golgi apparatus to the MHC-II compartment or APC membrane. Endocytosed antigens and Ii-MHC-II are degraded by proteases in the endosome. The class II-associated invariant chain peptide (CLIP), initially occupying the peptide-binding groove of MHC-II, is replaced by a longer antigenic peptide with the aid of HLA-DM. Peptide-MHC-II complexes are then transported to the APC membrane to activate CD4^+^ T cells, which differentiate into T helper (Th), regulatory T (Treg), or follicular helper T (Tfh) cells. **(B)** Naive lymphocytes reside in the WP, particularly in subregions such as TCZs, BCZs, and BCs. **(C)** Endogenous or exogenous antigens in the blood enter the MZ or surrounding RP via terminal arterioles. These antigens are captured and phagocytized by migratory DC (mDC) or Mφ, which process them into APCs. APCs then transport antigens to TCZs to activate CD8^+^ T cells (cellular immunity). Simultaneously, MZBs capture antigens and generate GCs in the BCZs, initiating humoral immunity. **(D)** Cytotoxic T cells subsequently migrate out of the WP through the BC and exit the spleen.

Splenic DCs, comprising approximately 3% of all CD45^+^ cells in the spleen, originate from BM progenitors and serve as “professional” APCs in adaptive immunity (Figures 2 and 3). These DCs include two classical subsets along with interferon-producing plasmacytoid DCs (pDCs). One major subset, cDC1s (CD8α^+^CD11b^-^) is primarily found in the TCZ, where they uptake dying cells and cross-present antigens to CD8^+^ T cells 23. Most splenic cDC1s express XCR1 and CD8αα, with distinct subpopulations residing in the WP, where they express DEC205, or in the MZ and RP, where they express CD103 or Langerin 24. The second major splenic DC subset, cDC2s (CD8α^-^CD11b^+^), is primarily localized to the RP and MZ, where they present MHC-II-peptide complexes to CD4^+^ T cells. At steady state, SIRPα^+^ CD11b^+^ cDC2s localize to the BC and consist of two subpopulations. ESAM^hi^ cDC2s depend on NOTCH2 and RBPJ signaling for development, express ESAM, CD11b, CD4, and DCIR2 (33D1), and are specialized for robust CD4^+^ T cell activation and antibody responses 25. In contrast, ESAM^lo^ cDC2s express CX3CR1, CD11b, and 33D1 but lower CD4 levels. They are NOTCH2- and IRF4-independent, secrete inflammatory cytokines such as TNF-α and IL-12, and share some characteristics with monocytes and macrophages 26. Additionally, pDCs, a non-classical DC subset, arise from both dendritic and IL-7R^+^ lymphoid progenitors. Upon activation, pDCs secrete large amounts of type I interferons, IL-12, and IL-18, which enhance NKT cell activity, boost effector CD8^+^ T cell responses, and drive CD4^+^ T cell polarization toward a Th1 phenotype 27.

Traditionally, blood-derived monocytes are thought to migrate into the MZ in response to pathogens or chemokines, where they stimulate T cell-independent MZB cell responses 28. However, this paradigm has been challenged by findings that undifferentiated monocytes, including CX3CR1^int^Ly6C^hi^ and CX3CR1^hi^Ly6C^lo^ subsets, accumulate in the spleen under steady-state conditions, outnumbering those in circulation. Intravital imaging of the spleen reveals that monocytes form clusters of approximately 20-50 cells in the subcapsular RP, particularly near venous sinuses and collecting veins at the organ's periphery 29. These splenic monocytes retain the ability to differentiate into diverse myeloid subsets, such as non-conventional DCs and Mφs. Notably, a specific subset of monocytes in the RP differentiates into CD11c^+^ “TIP (TNF-iNOS)-DCs”, which are highly inflammatory, producing TNF-α and nitric oxide (NO). Although these cells are inefficient APCs for naive T cells, they can reactivate effector or memory T cells and NK cells 30. Furthermore, the spleen acts as a reservoir for undifferentiated monocytes, marked by CX3CR1 with varying levels of Ly6C, which can be mobilized to other organs during inflammatory responses, supplementing monocytes released from the BM 13.

**Figure 4:**
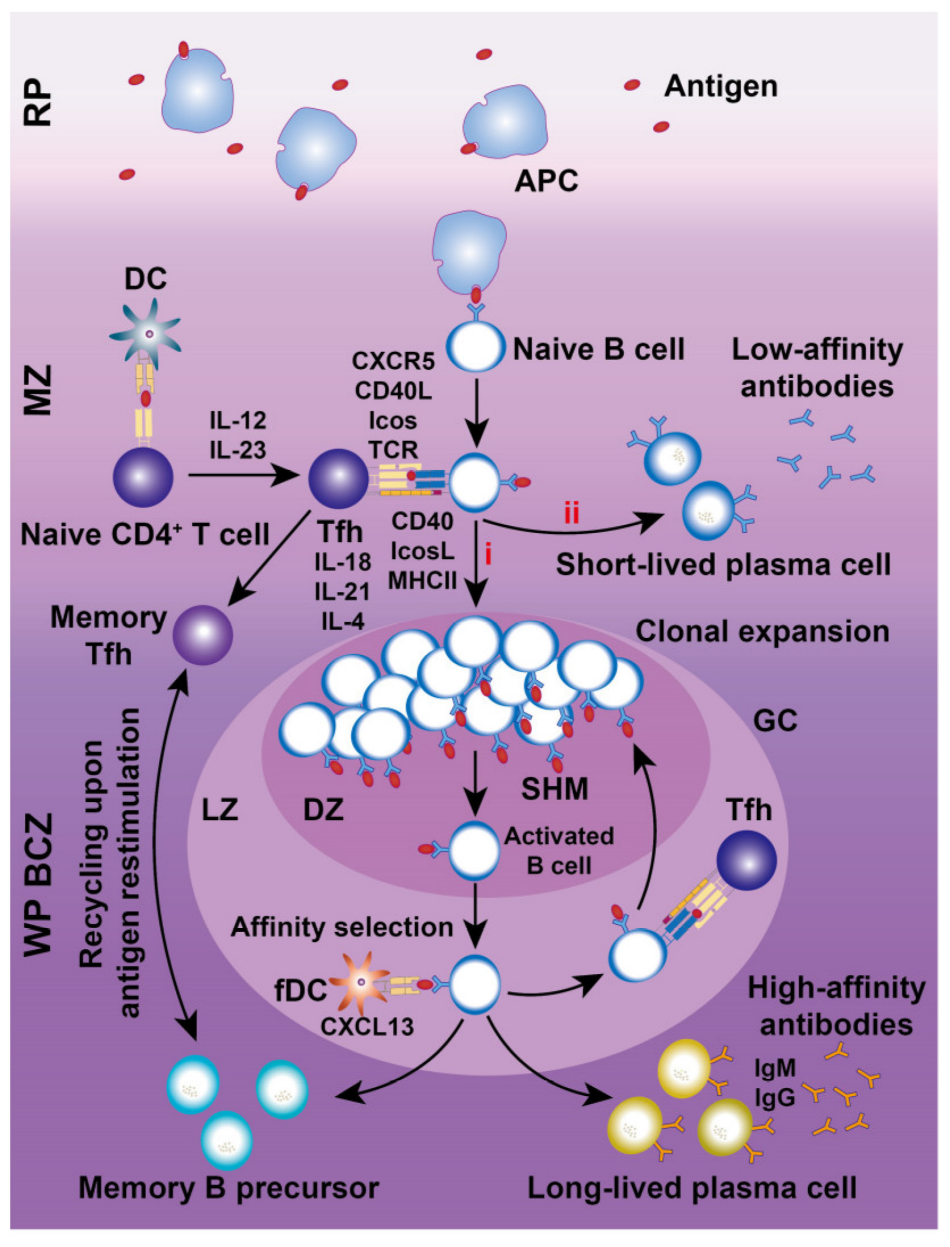
Schematic illustration of humoral immune responses in the spleen. Antigens are first captured by APCs in the RP and subsequently presented to naive B cells in the MZ. Most antigen-carrying naive B cells migrate into the GC within the BCZ for differentiation into GC B cells (i) undergoing somatic hypermutation (SHM) with the assistance of follicular Tfh cells or into short-lived plasma cells (ii). Activated B cells are proliferated in the DZ of the GC before migrating into the bright zone (BZ), where fDCs perform “affinity selection”. B cells with low-affinity are directed back to the DZ for further rounds of SHM, guided by interactions with fDCs and Tfh cells. Conversely, B cells with high-affinity receptors successfully exit the GC, differentiating into memory B cells or long-lived plasmablasts, which secrete large quantities of high-affinity antibodies against pathogens. In contrast, clones with weaker affinities gradually undergo apoptosis.

Chemokines are essential for recruiting and retaining B and T cells in their respective zones. CXCL13 attracts CXCR5^+^ B cells to follicular BCZs, while CCL19 and CCL21 recruit CCR7^+^ T cells and antigen-presenting DCs to TCZs 31, 32. Certain B cell lineages express S1PR1 and S1PR3, which bind to the lysophospholipid S1P, triggering chemotactic responses and promoting their accumulation in the MZ and RP 33. MZBs shuttle continuously between the MZ and follicles through transient desensitization and resensitization of S1PR1 34. Splenic follicular B cells (FOBs) also rely on S1PR1 for transit from follicles to the MZ and subsequent egress from the spleen via the RP 34. Although FOBs primarily participate in T cell-dependent immune responses, MZBs reside between the MZ and RP, capturing blood-borne antigens via complement receptors and facilitating both T cell-independent and -dependent immune responses. With regard to humoral immunity (Figure 4), MZBs bind antigen-carrying APCs, internalize antigens, and migrate to the follicle border via chemokine signaling. There, they interact with CXCR5^+^ Tfh cells, facilitating FOB somatic hypermutation, proliferation, differentiation, and maturation 35.

CD4^+^ and CD8^+^ T cells, the key effectors of the adaptive immune system, segregate within the PALS of the murine WP at steady state and after systemic infection 6. CD4^+^ T cells primarily localize at the outer PALS border near B cell follicles. Tfh cells within the TCZ/PALS support B cells in producing high-affinity antibodies through cytokine secretion (BCL-6, IL-6, IL-21) and co-stimulatory interactions (ICOS-ICOSL and CD40-CD40L; Figure 4) 36. During immune activation, Tfh cells upregulate CXCR5 to migrate to the T-B border, while B cells increase CCR7 to relocate from the BCZ 37. In contrast, naive CD8^+^ T cells are primarily confined to the central PALS of the WP. Upon activation, primed cytotoxic T cells exit the WP via the BC, migrate to the MZ and RP, and eliminate pathogens (Figure 3) 12. A subset of memory CD8^+^ T cells returns to the PALS, while CD62L-CXCR3^+^ memory CD8^+^ T cells remain in the RP 38. These observations highlight the pivotal role of chemokines in directing T cell positioning in relation to antigen presentation and inflammatory cytokines within the spleen.

NK cells predominantly reside in the RP but migrate to the WP during immune responses, where they produce IFN-γ to support T cell polarization and enhance early innate immunity by promoting DC differentiation 39. NK cells are critical for defense against viral infections, tumors, and autoimmune diseases. Although traditionally classified as innate immune cells, NK cells exhibit adaptive-like characteristics, including pathogen-specific expansion, the formation of memory-like cells, and enhanced secondary responses upon re-exposure 40. Intravital microscopy studies reveal that splenic NKT cells are primarily located in the MZ and RP, where they interact with MZBs to detect blood-borne antigens and initiate activation 16. These findings highlight the importance of spatial organization within the spleen, as B cell compartmentalization plays a critical role in regulating T and NKT cell activation, antigen presentation, and the shaping of adaptive immune responses.

#### 2.2.2 Cell recruitment to the spleen

Beyond the circulating immune cells that continuously migrate through the spleen under homeostatic conditions, disease states can drive the recruitment of additional immune populations. Auffray *et al.* 41 reported that *Listeria monocytogenes* infection mobilizes CX3CR1^int^Ly6C^hi^ monocytes from the BM into circulation via CCR2, followed by their CX3CR1-dependent accumulation in the splenic MZ and TCZs. Once there, these monocytes differentiate into DC-like cells, known as TNF-α- and inducible nitric oxide synthase-producing Tip-DCs. *Listeria* infection also recruits NK cells to the spleen through CCR5 and MyD88-dependent signaling, where they produce IFN-γ to promote monocyte maturation into Tip-DCs. Additionally, bacterial infection can recruit innate response activator B cells to the RP, where they reside via VLA-4 and LFA-1 adhesion and secrete GM-CSF to regulate innate immunity 42. The recruitment dynamics vary depending on the pathogen's type and characteristics. Norris *et al.* 43 demonstrated that the Armstrong strain of lymphocytic choriomeningitis virus induces transient splenic accumulation of monocytes and NPs, while the chronic Clone 13 (C13) strain drives sustained recruitment of these cells that acquire characteristics resembling MDSCs, which potently suppress virus-specific T cell immunity.

Recruitment of numerous cell populations has also been documented in individuals suffering from non-bacterial diseases. For instance, BM-derived CD45^+^Col^+^ fibrocyte cells, functioning in antigen presentation and priming of CD8 T cell responses, exhibit recruitment to spleen to facilitate innate and adaptive immune responses to hepatotoxic injury, renal fibrosis, or lipopolysaccharide (LPS)-induced inflammation 44. Similarly, tumor-bearing mice exhibit thymus-derived T cell recruitment to the spleen, a process regulated by prostaglandin E2 (PGE2) levels 45. Immunosuppressive populations, including MDSCs and NPs, are also recruited to spleen under various pathological conditions such as polymicrobial sepsis, LPS-induced lung injury, and breast cancer 1, 46. This dual recruitment of pro-inflammatory and immunosuppressive cell populations to the spleen presents a complex interplay that can either enhance or compromise host immunity. Therefore, the relative contributions of these recruited cells to immune regulation need to be further studied and analyzed in terms of specific disease progression.

#### 2.2.3 Cell amplification in the spleen

Splenocytes can self-proliferate and renew, contributing to both local and systemic immune homeostasis and aiding in disease prevention. A defining feature of adaptive immunity is the clonal expansion of antigen-specific T and B cells, generating a large pool of short-lived effector cells alongside a smaller subset of long-lived memory cells. For example, Natalini *et al.* 47 utilized cytofluorometric methods to confirm the high proliferative potential of T cell subsets, and B cells occurred within GCs. Under normal conditions, most splenic T cells remain quiescent, with a fraction dividing in response to environmental antigens or cytokine-driven homeostatic signals 48. Naive FOBs reside within WP follicles but, upon activation, cycle rapidly between the LZ and DZ of the GC under the influence of CXCR5 and CXCR4 signaling, supporting rapid proliferation, somatic hypermutation, and differentiation 49. CD4^+^ Tfh cells are essential for GC formation and the production of long-lived, high-affinity B cells, while regulatory CD4^+^ (T_FR_) and CD8^+^ T cells co-localize with Tfh cells to limit excessive GC responses 50.

Elchaninov *et al.* 51 revealed that allogeneic subcutaneous transplantation of splenic fragments in mice promotes structural recovery within 30 days, including the reconstitution of monocyte-macrophage, megakaryocyte, and B lymphocyte populations. This process appears to be driven by circulating hematopoietic stem and progenitor cells (HSPCs) replenishing the splenic cellular milieu as regulated by GM-CSF, IL-1β, IL-3, CXCL12, G-CSF, LIF, TNF-α, c-Kit, CXCR2/4 and the transcription factor C/EBPβ 52. For example, Wang *et al.* 53 and others 54 demonstrated that bacterial infections, particularly those involving *Akkermansia muciniphila*, activate and mobilize HSPCs from the BM to the spleen via TLR2/4 and MyD88/TRIF signaling pathways. Furthermore, Wang *et al.* 55 identified that HSPCs are retained in the splenic RP through interactions between VCAM-1-expressing Mφs and VLA-4-positive HSPCs. Splenic hematopoiesis has been observed in various disease models, such as cancer 52, atherosclerosis 56, and rheumatoid arthritis 57, where BM-derived splenic HSPCs often display a myeloid-biased differentiation at the expense of erythropoiesis and lymphopoiesis. Importantly, splenic HSPCs also contribute to immune modulation by replenishing the immune cell repertoire. Bono *et al.* 58 showed that during *Candida albicans* infection, HSPCs transiently migrate to the spleen to generate trained Mφs that are primed for myeloid cell production and secrete elevated levels of proinflammatory cytokines upon re-exposure. Additionally, Ghosh *et al.* 59 identified an atypical HSPC population (LSK^-^ phenotype) that proliferates and differentiates into mature FOBs during non-lethal *Plasmodium* infection, subsequently participating in GC reactions and maturing into memory B cells or antibody-secreting cells.

#### 2.2.4 Cell mobilization from the spleen

During stress, disease onset, or immune activation, the spleen mobilizes cellular constituents to the nidus, exerting both beneficial and detrimental effects. In conditions such as ischemic myocardial injury, atherosclerosis, and infection-related diseases, splenic monocytes exhibit increased motility, entering circulation and migrating to lesions, where they differentiate into Mφs or DCs 60, 61. Mφs aid tissue repair by phagocytizing debris, producing inflammatory cytokines, and restoring homeostasis, while DCs process antigens and activate T cells. However, in cancers, splenic monocytes, particularly the CX3CR1^int^Ly6C^hi^ subtype, can migrate to the tumor microenvironment and differentiate into tumor-associated Mφs (TAMs) 62, which exacerbate cancer progression and serve as prognostic markers. Similarly, our research demonstrated that CD11b^+^CD43^hi^Ly6C^lo^ splenic monocytes migrate to the liver and differentiate into Mφs, contributing to liver fibrosis 63. Experimental stroke models also show splenic Mφ mobilization into circulation, with subsequent accumulation in the ischemic brain, exacerbating neurodegeneration 64. These findings highlight the context-dependent role of Mφ responses, which can either support tissue repair or drive disease progression. Beyond monocytes, the spleen also mobilizes other immune cells, including CD8^+^ T cells, B cells, NK cells and Treg cells, to alter host organ immunity 65, 66.

Targeting the mobilization of specific splenic immunocytes offers a promising therapeutic approach for disease modulation. Mesenchymal stem cell-derived extracellular vesicles (EVs) enhance neovascularization in irradiated tissues by increasing pro-angiogenic factor production, recruiting vascular progenitor cells, and mobilizing splenic monocytes to injury sites, thereby exhibiting pro-inflammatory potential 67. Similarly, Akbar *et al.* 68 reported that endothelium-derived EVs promote splenic monocyte mobilization in myocardial infarction by downregulating plexin-B2 and upregulating ITGB2. Moreover, pharmacological agents such as pegfilgrastim facilitate the migration of splenic activated monocytes and granulocytes to tumor sites, serving as effective adjuvants in monoclonal antibody-based immunotherapy 69. Altogether, the dynamic mobilization of splenic immunocytes is strongly associated with disease progression and therapeutic outcomes.

## 3. Neuro-immune interplay alters splenic immunocompetence in disease progression and treatment

### 3.1 Innervation of the spleen

Splenic nerves are primarily located around the perivascular adventitia and near immunocytes exerting both innate and adaptive immunity 3. Although Wu *et al.* 70 identified a network of nociceptive sensory fibers that enhance GC responses and humoral immunity via the CGRP-CALCRL/RAMP1 signaling axis, most studies demonstrated that sympathetic catecholaminergic nerves innervate the spleen but with absence of parasympathetic, sensory, or vagal innervation 3, 71. Nerve density is higher in the WP, especially around central arteries, than in the RP, where arterioles and capillaries predominate 4. The splenic reticular system forms non-endothelial vascular spaces harboring free-moving immunocytes, such as T and B lymphocytes, DCs, Mφs, and NK cells, allowing extensive neuro-immune interactions 13.

**Figure 5:**
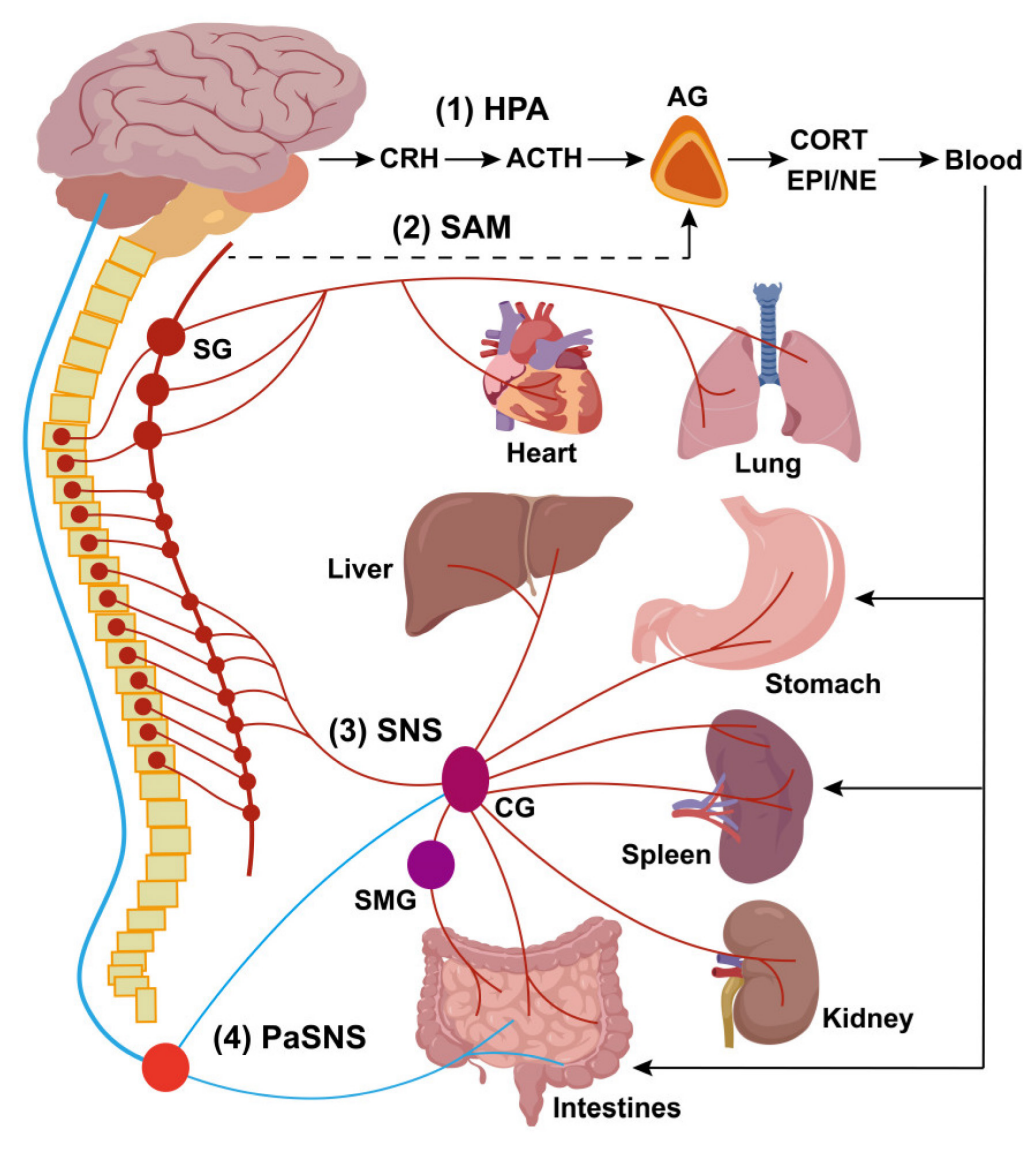
Schematic diagram depicting major neuroendocrine and autonomic pathways regulating secondary immune organs. This diagram illustrates the primary neuroendocrine and autonomic pathways involved in the regulation of secondary immune organs. The neuroendocrine pathways include: (1) The hypothalamic-pituitary-adrenal (HPA) axis, and (2) The sympatho-adrenal medullary (SAM) axis. The autonomic pathways consist of two “hardwired” circuits: (3) The sympathetic nervous system (SNS), and (4) The parasympathetic nervous system (PaSNS). In the HPA axis, corticotropin-releasing hormone (CRH) is secreted by hypothalamic neurons that project to the anterior pituitary. CRH stimulates the release of adrenocorticotropic hormone (ACTH), which in turn triggers the secretion of corticosterone (CORT) into the bloodstream. Circulating CORT has systemic effects, including modulation of secondary immune organs such as the spleen, LNs, and gut-associated lymphoid tissue (GALT). In the SAM axis, preganglionic sympathetic neurons release acetylcholine (Ach) in the adrenal medulla, stimulating the release of catecholamines, predominantly epinephrine (EPI) and, to a lesser extent, norepinephrine (NE), into the bloodstream. These circulating catecholamines (e.g., CORT in the HPA axis) have widespread effects, potentiating the activity of sympathetic nerves by activating adrenergic receptors in visceral organs. The SNS circuit to the spleen and LNs operates as a two-neuron chain. Preganglionic cholinergic sympathetic neurons synapse with postganglionic neurons, whose terminals innervate the spleen and LNs. Postganglionic neurons primarily release NE, which binds to adrenergic receptors expressed on immune cells, vasculature, and connective tissue in secondary lymphoid organs. In the spleen, the predominant adrenergic receptor subtype is the β2-adrenergic receptor (β2AdrR), and its activation regulates the immune cells' responses. Similarly, the PaSNS also follows a two-neuron chain structure. Preganglionic neurons, originating in the brainstem (medulla), exit the CNS as cranial nerves and synapse on postganglionic neurons embedded in visceral organs, particularly in the gut. Although there is no conclusive evidence that the PaSNS directly innervates the spleen, the vagus nerve influences splenic immune responses indirectly, likely through its connections to the celiac ganglion (CG), where vagal and sympathetic preganglionic fibers converge. Abbreviations: SG, sympathetic ganglia (also classified as inferior cervical ganglion); SMG, superior mesenteric ganglion; AG, adrenal gland.

The disease occurrence and progression in the body are closely monitored and modulated through the neuro-immune axis. The innervation of secondary immune organs, such as the spleen, mainly involves both neuroendocrine and autonomic pathways. The neuroendocrine pathways include the hypothalamic-pituitary-adrenal (HPA) axis and the sympatho-adrenal medullary (SAM) axis, while the autonomic pathways are mediated by the sympathetic (SNS) and parasympathetic nervous systems (PaSNS; Figure 5).

With regard to HPA axis, appropriate activation of corticotropin releasing hormone (CRH) neurons in the paraventricular nucleus of the hypothalamus and/or the central nucleus of the amygdala (PVN/CeA) regulates splenic humoral immune defenses. Bassi *et al.* 71 demonstrated that optical stimulation of CRH neurons in the PVN and CeA increases splenic nerve discharge, indicating a direct functional connection. Stressors can also activate the SAM axis, in which preganglionic sympathetic neurons secrete acetylcholine (Ach) in the adrenal medulla, triggering the release of catecholamines, predominantly epinephrine (EPI) and norepinephrine (NE), which can enhance sympathetic nerve activity by activating adrenergic receptors in immune organs. Kim *et al.* 72 showed that electro/chemical acupuncture stimulates the SAM axis, leading to catecholamine release that modulates inflammatory responses via β-adrenoceptor activation on immune cells.

The autonomic nervous system (ANS) is a major allostatic regulator essential for normal immune responses in the spleen and other primary/secondary lymphoid organs. The SNS circuit to the spleen and LNs is composed of a two-neuron chain: preganglionic cholinergic sympathetic neurons innervate postganglionic nerve terminals that terminate in the spleen and LNs. During neuro-immune interactions, NE acts on β2-adrenergic receptors (β2AdrRs) on splenic cells, modulating immune responses to maintain homeostasis 73. Additionally, the PaSNS is also a two-neuron chain of efferent nerves that supply the viscera, in particular the gut. Preganglionic neurons originate in the medulla, exit the central nervous system (CNS) as cranial nerves, and terminate on postganglionic neurons within target organs (depicted in Figure 5 is the parasympathetic supply to gut-associated lymphoid tissue (GALT)). Although no definitive evidence demonstrated PaSNS directly innervating the spleen, the vagus nerve appears to influence splenic immune responses, likely through the migration of vagally-modulated immune cells from the gut to the spleen 73. The vagus nerve regulates sympathetic activity in the spleen via the celiac ganglion (CG), where vagal and sympathetic preganglionic fibers converge. It enhances the anti-inflammatory effects of the efferent sympathetic response through α7 nicotinic acetylcholine receptor (α7nAChR)-positive memory T lymphocytes, which, characterized by high CD44 and low CD62L expression, secrete ACh that counteracts the sympathetically mediated suppression of TNF-α secretion, likely by binding to presynaptic α7ChR on sympathetic nerve terminals 74. Additionally, another vagal-sympathetic regulatory pathway has been described, wherein vagal sensory afferents are activated by catecholamines (EPI and NE), providing a centrally mediated negative feedback mechanism that adjusts sympathoadrenal activity 75. Circulating EPI, and to a lesser extent NE, secreted by the adrenal glands, reinforces neural stimulation of adrenergic receptors on immunocytes and vasculature within the spleen, facilitating a systemic stress response. Furthermore, some studies suggest that vagal stimulation may regulate the spleen through a more complex C1 neurons-SNS-splenic nerve-spleen-kidney axis, further highlighting the intricate neural-immune interactions governing splenic function 76.

In this review, we focus on the relationship between the ANS and splenic immunity, as recent studies (discussed in Part 5) have highlighted how physical stimulation of the cervical, vagus, or splenic nerves can modulate splenic immunity to treat various diseases. Evidence overwhelmingly points to bidirectional interactions between the ANS and the immune system, particularly the spleen, in regulating systemic inflammation and tumor immunity 73. The autonomic circuitry of the spleen is closely tied to the body's response to suffer or escape from potentially damaging and life-threatening pro-inflammatory “cytokine storms” resulting from systemic infections or unresolved immune responses in inflammatory diseases 77.

### 3.2 Functional neuroanatomy in communication with the immune system

**Figure 6:**
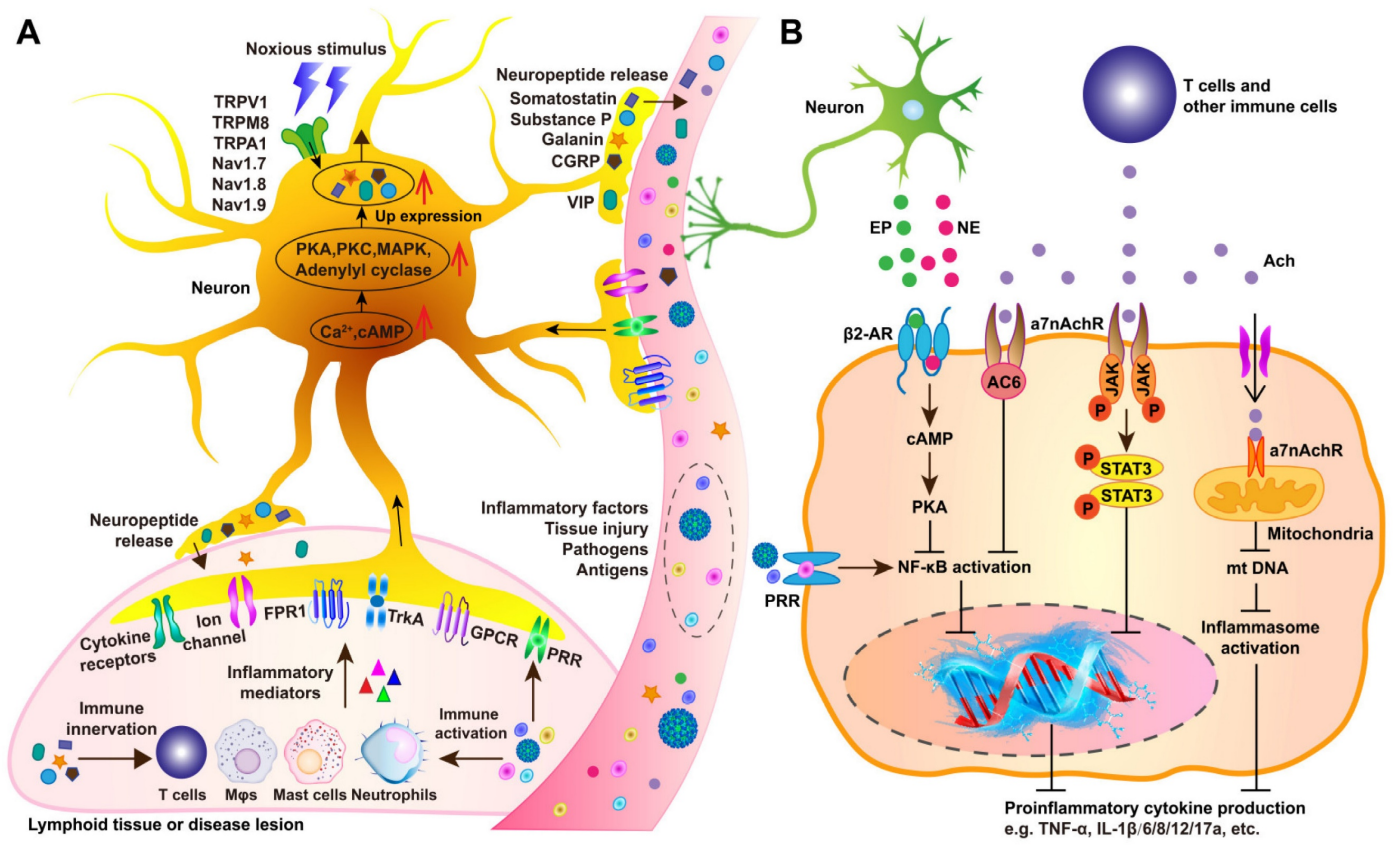
Molecular mechanisms underlying neuro-immune interactions. **(A)** Circulating or local pathogens, antigens, inflammatory factors, and tissue injury trigger the release of inflammatory mediators, such as proinflammatory cytokines (TNF-α, IL-1β, IL-6, and IL-17), to activate sensory neurons in the affected area by interacting with specific receptors, including cytokine receptors, G protein-coupled receptors (GPCRs), and tyrosine kinase receptor type 1 (TrkA). Additionally, pathogen-associated molecular patterns (PAMPs) can stimulate sensory neurons via pattern recognition receptors (PRRs), such as neuronal TLR4. Certain pathogens, such as *Staphylococcus aureus*, directly provoke nociceptors by secreting N-formyl peptides and α-hemolysin, which bind to formyl peptide receptor 1 (FPR1) or activate ion channels. Subsequent activation of secondary messengers, such as Ca²⁺ and cAMP, leads to the activation of intracellular kinases, including adenylyl cyclase, protein kinase A (PKA), protein kinase C (PKC), and mitogen-activated protein kinase (MAPK). This signaling cascade likely generates action potentials and lowers the activation threshold of nociceptive receptors. These include transient receptor potential cation channels (TRPV1, TRPA1, and TRPM8) and voltage-gated sodium channels (Nav1.7, Nav1.8, and Nav1.9), amplifying the response to noxious stimuli. Furthermore, activated neurons release neuropeptides, such as calcitonin gene-related peptide (CGRP), galanin, somatostatin, substance P (SP), and vasoactive intestinal peptide (VIP). These neuropeptides influence immune responses through an axon reflex mechanism.** (B)** Ach and NE modulate cytokine release by immune cells in response to inflammation and noxious stimuli. Ach binds to α7 nicotinic acetylcholine receptors (α7nAChRs) on Mφs and other immune cells, activating intracellular signaling pathways, including adenylyl cyclase 6 (AC6) and JAK2/STAT3, to suppress the release of proinflammatory cytokines. Additionally, extracellular ATP promotes Ach influx, enabling mitochondrial α7nAChR activation, which reduces mitochondrial (mt) DNA release. This, in turn, inhibits inflammasome activation and subsequent inflammatory responses. Similarly, NE and EPI bind to β2AdrRs on Mφs and other immune cells, triggering intracellular signaling cascades involving cAMP and PKA, which suppress nuclear factor-κB (NF-κB) activation and the secretion of proinflammatory cytokines.

Generally, afferent (sensory) neurons apperceive immune cell activity and modulate immune responses, while efferent autonomic (motor) neurons serve as key regulators of immunity. Together, afferent and efferent circuits form reflexive systems that control immune responses and inflammation. Sensory neuron activation often results from peripheral immunocyte activation alongside with secretion of cytokines and signaling molecules. Immune activation occurs in response to pathogens or sterile tissue injury via pathogen-associated molecular patterns (PAMPs) or damage-associated molecular patterns (DAMPs) engaging PRRs (Figure 6A) 78. This triggers intracellular pathways, including NF-κB, AP-1, and inflammasomes, leading to the production of pro-inflammatory cytokines (TNF, IL-6, IL-1β), chemokines, and immune mediators. These molecules promote vasodilation, vascular permeability, and leukocyte recruitment while also directly affecting sensory neurons at infection or injury sites, altering CNS signaling. NPs, Mφs,Beyond immune detection, sensory neurons actively regulate immunity and inflammation. Studies on inflammatory bowel disease (IBD), arthritis, asthma, skin inflammation, chronic itch, and bacterial infections show that sensory neurons release neuropeptides, such as substance P (SP), CGRP, and vasoactive intestinal peptide, which interact with endothelial cells, NPs, Mφs, and other immune cells to modulate local immune responses 79.

ANS-driven immune modulation in organs like the thymus, BM, spleen, LNs, and GALTs plays a vital role in neuroimmune circuit during conditions such as sepsis, IBD, arthritis, obesity, and cancer 80, 81. Experimental evidence confirms the involvement of efferent autonomic fibers, both sympathetic and vagus nerves, in reflexive immune and inflammatory regulation. Catecholamines such as NE and EPI, released by sympathetic postganglionic fibers and the adrenal medulla, regulate immune cell functions via adrenergic receptors on NPs, monocytes, Mφs, T cells 82. Catecholaminergic regulation through β2-adrenergic receptor (β2AdrR) signaling activates the β2AdrR-cAMP-PKA pathway, thereby suppressing NF-κB translocation, reducing pro-inflammatory cytokines (e.g., TNF and IL-12), and increasing anti-inflammatory mediators (e.g., IL-10 and TGF-β) (Figure 6B) 82. In contrast, α-adrenergic receptor signaling in monocytes and Mφs promotes TNF and other pro-inflammatory cytokines 83. Sympathetic nerve fibers co-localize with T cells, Mφs, and B cells in the splenic MZ, facilitating immune cell entry. Catecholaminergic innervation is particularly dense in TCZs and areas containing mast cells and Mφs, while BCZs remain sparsely innervated 84. This architectural organization facilitates efficient catecholaminergic regulation within the spleen.

Cholinergic signaling along the efferent vagus nerve also exerts potent anti-inflammatory functions, collectively known as cholinergic anti-inflammatory pathway (CAP), in endotoxemia, sepsis, arthritis, IBD, hemorrhagic shock, postoperative ileus, and renal ischemia-reperfusion injury 85. Ach, the primary vagal neurotransmitter, dose-dependently suppresses TNF, IL-1β, IL-6, and IL-8 secretion from Mφs by regulating the α7nAChR signaling to mediate NF-κB inhibition, JAK2/STAT3 activation, and inflammasome modulation. (Figure 6B). Functional interplay between the efferent vagus nerve and the splenic nerve underpins the inflammatory reflex. Vagal fibers from the dorsal motor nucleus (DMN) innervate the CG and superior mesenteric ganglia, which, in turn, control splenic catecholaminergic neurons 86. Vagus nerve stimulation elevates Ach levels in the spleen, where catecholaminergic terminals are in proximity to T cells expressing choline acetyltransferase (ChAT), the enzyme required for Ach synthesis, and βAdrRs. Splenic nerve catecholaminergic output through βAdrR signaling activates ChAT^+^ T cells to secrete Ach, which then interacts with α7nAChRs on Mφs and other immune cells to suppress TNF and other proinflammatory cytokines.

Parasympathetic (vagal) and sympathetic signals synergistically regulate splenic innate and adaptive immunity. Vagus nerve stimulation reduces circulating TNF levels and mitigate renal ischemia-reperfusion injury via α7nAChR signaling in the spleen 87. Moreover, intervention of the cholinergic-sympathetic pathway has been implicated in regulating splenic B cell antibody production during* Streptococcus pneumoniae* infection, T cell activation in experimental hypertension, and immune cell mobilization 88. Additionally, Mφs, DCs, T cells, and other immune cells express ionotropic, metabotropic, and G-protein-coupled receptors for various neurotransmitters. In addition to Ach and NE, other neurotransmitters, such as dopamine, serotonin (5-hydroxytryptamine, 5-HT), SP, γ-aminobutyric acid (GABA), and glutamate also contribute to neuroimmune interactions, thus modulating splenic immunity 78, 87.

## 4. Genetic and chemical immunomodulation of the spleen for disease treatment

**Figure 7:**
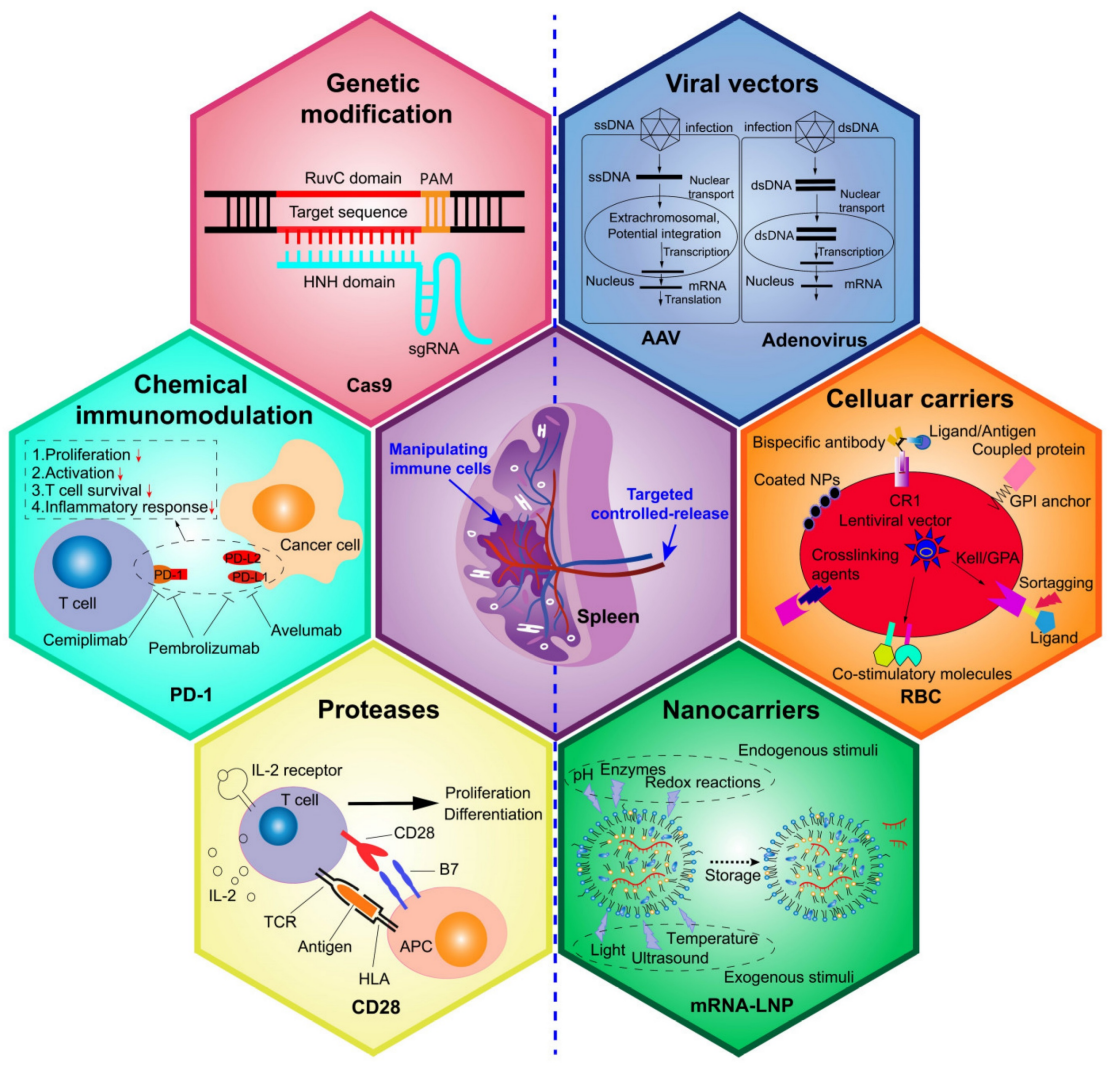
Schematic diagram of genetic and chemical immunomodulation of the spleen and targeted controlled-release of allogenic materials. This schematic illustrates two key aspects: (1) Representative approaches for splenic immunomodulation include the gene editing such as CRISPR technology to splice or insert target genes in the host genome, the chemical agents such as avelumab, pembrolizumab, and cemiplimab to disrupt the PD-1/PD-L1 immune checkpoint interaction, and the protein preparation such as IL-2 protein reagents to stimulate T-cell proliferation and differentiation. (2) Techniques for spleen-targeted delivery include viral vectors such as AAV and adenovirus for gene insertion, engineered cellular carriers (such as red blood cells) to transport diverse therapeutic substances, and artificial nanocarriers designed for the controlled release of allogenic materials under specific conditioned stimuli.

Although previous studies have suggested splenectomy as a strategy to mitigate disease progression, mounting evidence has highlighted the severe adverse effects associated with this approach 89. Consequently, researchers have shifted focus toward genetic and chemical strategies to modulate splenic immunity and microenvironment as a means to address dysregulated immunity, enhance immune defenses, and improve disease outcomes (Figure 7). Table 1 provides an overview of various therapeutic strategies targeting the spleen's role in immune regulation, including their mechanisms, reported efficacy, and potential side effects.

### 4.1 Genetic modification of splenocytes to enhance disease-fighting immunity

Genetic interventions, including the inserting, editing, or silencing of specific genes in splenic lymphocytes, show significant potential to activate and enhance the splenic immune system, enabling more effective responses to disease. Introducing specific nucleotide sequences into splenocytes often aims to express target proteins that modulate host immunity. For example, Kranz *et al.* 90 developed three RNA vaccine lipoplexes, targeting splenic DCs for delivery, of which the HA-LPX induced robust proliferation of HA-specific T-cell receptor (TCR)-transgenic CD8^+^ and CD4^+^ T cells in the blood, LNs and spleen, the gp70-LPX triggered fully functional antigen-specific T cells, representing 30-60% of total CD8^+^ T cells, while the OVA-LPX promoted profound CD8^+^ T cell expansion and memory cell formation. Similarly, Kurosaki *et al.* 91 developed γ-PGA-coated complexes containing the pUb-M DNA vaccine vector, which demonstrated selective and efficient transgene expression in the splenic MZ, significantly inhibiting melanoma growth and metastasis.

Gene editing or silencing techniques, such as CRISPR and small interfering RNA (siRNA), have also been employed to modify gene expression in splenocytes for therapeutic purposes. He *et al.* 92 reported that double knockout of the Per1/Per2 genes impaired splenic immune function in 14-month-old mice, reducing lymphocyte tolerance to oxidative stress and compromising the ferroptosis defense system, thereby diminishing splenic immune activity. Additionally, Ding *et al.* 93 found that silencing MyD88 expression in HD11 cells within spleen significantly suppressed the production of inflammatory cytokines, including IL-1β, TNF-α, IL-8, NF-κB, and TLR4, and mitigated LPS-induced inflammatory responses. Leuschner *et al.* 94 developed an optimized lipid nanoparticle delivering CCR2-silencing siRNAs to splenic monocytes, in which efficient degradation of CCR2 mRNA reduced infarct size after coronary artery occlusion, prolonged normoglycemia in diabetic mice following pancreatic islet transplantation, and decreased tumor volumes TAMs.

Despite the immense potential of spleen-targeted genetic immunomodulation, several challenges remain in practice 110. First, genetic modification is a highly personalized therapeutic strategy that requires precise identification of target genes associated with the specific disease pathogenesis. Second, it demands advanced technical expertise for both gene editing and the spleen-targeted delivery of exogenous nucleic acids. Finally, individual variability in genetic and immunological responses presents an additional layer of complexity, which must be accounted for in clinical applications.

### 4.2 Chemical immunomodulation of the spleen for disease immunotherapy

Utilizing specialized drugs targeting specific molecules or signaling pathways in splenocytes has emerged as a promising therapeutic strategy. For instance, spleen-targeted JAK inhibitors and S1P receptor modulators have demonstrated efficacy in regulating immune cell activation and migration in inflammatory and autoimmune diseases 95, 96. Extensive research has investigated various chemical reagents and protease-based therapies to modulate splenic immunocyte activity. For example, protease inhibitors targeting spleen-resident macrophages have shown potential in controlling excessive immune activation in autoimmune disorders, while small-molecule immunomodulators have been explored to enhance antigen presentation in splenic DCs 105, 106.

Concretely, recent research has introduced a variety of chemical agents to modulate splenic immunity. Blocking intermediary components of the brain-spleen-brain cycle has been shown to reduce stroke-induced brain injury after administration with adrenergic receptor antagonists such as carvedilol or propranolol, which also possess the properties of reversing post-stroke immunosuppression, reducing spleen volume loss, decreasing cerebral infarction size, and potentially lowering susceptibility to bacterial infections 97. Spleen tyrosine kinase-targeted inhibitors such as fostamatinib (and its active metabolite R406), HMPL-523, and cevidoplenib have demonstrated efficacy in treating warm autoimmune hemolytic anemia, immune thrombocytopenia, cancers, vasculitis, arthritis, glomerulonephritis, acute lung injury, and even COVID-19-associated inflammation and coagulopathy 98. Moreover, various chemical reagents have been verified for their ability to modulate specific immunocyte subsets in the spleen. For example, 17β-estradiol has been shown to enhance the response of splenic B cells to TLR9 agonists in lupus models 99. Pioglitazone, by activating PPAR-γ, increased the proportion of CD4^+^CD25^+^Foxp3^+^ regulatory T (Treg) cells while reducing CD3^+^CD4^+^IFN-*γ*^+^ and CD3^+^CD4^+^IL-4^+^ inflammatory T cells in spleen, thereby mitigating Schistosoma japonicum-induced pathologies 100. Herbal ingredients such as licochalcone A, artemisinin, and Eucommia ulmoides extracts have been demonstrated to stimulate splenic T and B cell proliferation 101, 102. Additionally, various immunostimulants targeting Toll-like receptors (TLR2, TLR4, TLR5, TLR7, TLR8, and TLR9) on splenic T cells have shown robust immunomodulatory effects 103, 104.

Concurrently, protease-based treatments have emerged as practical and effective methods for modulating splenic immune responses. For instance, in tMCAO models, the application of αCD147 successfully eliminated the inflammatory activation of spleen-derived Ly6C^high^ monocytes/Mφs infiltrating the brain. Similarly, recombinant IL-33 regulated splenic T cell responses by inhibiting the Th1 response while promoting Treg cell activity, and RTL551/1000 prevented splenic atrophy, splenocyte mobilization, and pro-inflammatory immune cell infiltration into the mouse brain 97. In addition to mitigating immunosuppression through inhibitory pathways, activating costimulatory molecules to enhance splenic immunocyte activity and boost immune responses has gained significant attention. For example, a combination of the anti-GITR antibody (G3c) and the CD28 superagonist (D665) induced the generation of a substantial population of splenic CD4^+^Foxp3^-^ T cells capable of secreting high levels of IL-10 with potent immunosuppressive properties, effectively alleviating DSS-induced inflammation both systemically and locally 107. Similarly, treatment with an agonistic OX40 monoclonal antibody promoted the expansion of antigen-experienced effector CD8^+^ (CD11a^hi^CD44^hi^) cells and IFN-γ/TNF producing CD4^+^ T cells in the spleen, enhancing protective immunity following vaccination, as evidenced by an increased number of protected mice and delayed progression to blood-stage infection after challenge with wild-type sporozoites 108. Beyond these examples, numerous agonistic and antagonistic antibodies have been developed to modulate splenic immunocyte responses against pathogens. These include agents that inhibit immune checkpoint receptors such as CTLA-4, PD-1, and PD-L1, or activate costimulatory molecules such as CD27, CD28, CD40, and CD137, effectively unleashing T cell immunocompetence and amplifying immune responses.

Undeniably, experimental and clinical data indicate that chemistry immunomodulation of the spleen for disease immunotherapy is associated with potential side effects and limitations. Some drugs can directly cause splenomegaly by affecting splenic cells or indirectly as a consequence of disturbances in other organs, such as the liver, or systemic disruptions, including the haematoimmunological system 109. Furthermore, certain drugs have been reported to induce severe hemolytic anemia and thrombocytopenia, both of which are often linked to splenomegaly 109. Another contributing factor to splenic enlargement is venous congestion resulting from liver dysfunction, including portal vein occlusion as a secondary complication of drug treatment. Additionally, during administration, these therapies may lead to immune tolerance or provoke immune-related toxic reactions, further complicating their clinical application.

### 4.3 Controlled-release targeting the spleen

Efficient delivery of exogenous nucleic acids, proteases, or chemical reagents to the spleen is a critical factor in achieving effective immunomodulatory outcomes. The spleen is distinguished by its dual circulation pathways: open circulation, where arteries deliver blood directly to parenchymal cells, and closed circulation, where blood is delivered via splenic sinusoids containing pores ranging from 200 to 500 nm in size 111. Additionally, the spleen exhibits slower blood flow compared to other organs, which facilitates its accessibility for intravenously administered nanocarriers designed for controlled release of therapeutic substances 112. Controlled-release drug delivery systems targeting the spleen utilize both passive and active mechanisms to enhance therapeutic efficacy and minimize systemic side effects. Passive targeting takes advantage of the spleen's unique anatomical features, such as its fenestrated vasculature and slow blood flow, which facilitate the accumulation of nanoparticles within an optimal size range. Active targeting involves functionalizing drug carriers with ligands that specifically bind to receptors expressed on splenic cells, including CD169 on Mφs and DEC-205 on DCs, thereby promoting selective uptake by these immune cells 113.

A common strategy for spleen-targeted delivery involves using modified viruses as carriers to precisely transport genes to splenic cells. For instance, a non-replicating modified vaccinia virus Ankara (MVA) was engineered as rMVA-human IL-7-Fc, which enhanced the proportions of total and activated B, T, and NK cells, as well as myeloid subpopulations (e.g., Ly6C^high^, Ly6C^int^, and Ly6C^neg^ cells) in the spleen specifically, demonstrating potential as an immunotherapeutic agent in sepsis models 114. In addition, Bates *et al.* 115 characterized human adenovirus type 49 as an effective viral platform for *ex vivo* and *in vivo* transduction of lung and spleen cells through interactions with various surface molecules for entry. Currently, lentiviral vectors, retroviral vectors, adenoviral vectors, and AAVs are widely employed for gene transfection targeting diverse immune cells. However, despite their utility, these vectors exhibit strong immunogenicity and pose potential infection risks, highlighting the need for improved delivery platforms with enhanced safety profiles.

In comparison to viral approaches, nonviral methods are increasingly appealing for both gene and drug controlled-delivery. Traditional physical stimulation techniques, such as needle injections, ballistic pressure injections (gene gun), electric fields (electroporation), hydrodynamic pressure (water perforation), magnetic fields (magnetic transfection), and ultrasound (ultrasonic perforation), are limited by issues such as imprecision, potential cell damage, labor intensity, time consumption, and, in some cases, applicability restricted to superficial tissues 116. Recent advancements have focused on the development of diverse nanocarriers, designed to be activated by physical stimuli (e.g., ultrasound, photoradiation, magnetic induction, or physiological pH sensing) or to function independently of such triggers 117. These systems are gaining prominence due to their proven safety, effectiveness, and large capacity for packaging therapeutic components with varying molecular weights. For instance, Álvarez-Benedicto *et al.* 118 developed spleen selective organ targeted (SORT) lipid nanoparticles (LNPs) to deliver Cre recombinase mRNA and CAR-encoding mRNA to T cells in a lymphoreplete B-cell lymphoma model. This approach significantly facilitated the *in situ* generation of CAR T cells, reduced liver metastases, and prolonged survival. Similarly, Pan *et al.* 103 designed a nanocomposite sLNPs-OVA/MPLA for spleen-selective co-delivery of mRNA antigens and TLR4 agonist, which enhanced the efficacy of spleen-targeted mRNA vaccines by eliciting synergistic immunostimulation and robust Th1 immune responses. Numerous studies have also explored spleen-targeted nanocomplexes for controlled delivery of long or short exogenous nucleic acids, including mRNA, siRNA, plasmids, Cas9 mRNA/single-guide RNA, and Cas9 ribonucleoprotein complexes) to non-/specific splenocytes 110, 119. For targeted delivery of chemical therapeutics to the spleen, Kim *et al.* 120 developed a glycocalyx-mimicking platform capable of spleen-specific delivery of therapeutic cargoes without inducing toxicity. Additionally, Wei *et al.* 121 established ultrasound-responsive polymersomes for allogenic material delivery to any organ (including spleen) regardless of depth, for instance, enabling accelerated release of doxorubicin in any tumor nidus once upon ultrasound irradiation. To enhance targeting precision, researchers have conjugated nanocarriers with specific molecules, such as ligands, antibodies, or protein coronas, to focus delivery on the spleen or specific splenic cell populations 122. Targeted mRNA delivery to the spleen and its resident cell types represents a novel and promising approach in immunotherapy. For instance, conjugating CD4 antibodies to LNPs has been shown to facilitate specific mRNA delivery to splenic CD4^+^ T cells, achieving approximately 30-fold higher mRNA expression in these cells compared to non-targeted LNPs 123. Additionally, mannose-functionalized poly(β-amino ester) nanoparticles have demonstrated selective targeting of APCs within the spleen, thereby enhancing the efficacy of mRNA vaccines 124.

Moreover, the development of ionizable lipid nanoparticles has enabled *in vivo* engineering of CAR T cells by delivering mRNA directly to T cells in the spleen, offering a potential strategy for cancer immunotherapy 125. Furthermore, αCD3-targeted LNPs were shown to aggregate CD8^+^ T cells in the spleen, promote their migration from the WP to the RP, and drive their differentiation into memory and effector phenotypes 126. The strengths and challenges of various spleen-targeted nanomedicines are summarized in Table 2.

An emerging innovation in drug delivery involves the use of cells as transport vehicles. Among these, RBCs have been commonly proposed as carriers to enhance vascular and systemic delivery of drugs, either encapsulated within their intracellular volume or conjugated to their cellular surface. Several studies have explored RBCs as carriers for delivering antigens or nanoparticles to the spleen to modulate immune responses. For example, Wu *et al.* 127 developed a DNA vaccine-encapsulating polymeric nanoparticle system that was intentionally hitchhiked on the re-isolated RBCs. This strategy enabled preferential accumulation in the spleen, promoting neoantigen expression by APCs, enhancing robust neoantigen-specific T-cell immune responses, effectively preventing tumorigenesis in a personalized manner and slowing tumor growth in aggressive hepatocellular carcinoma models. Building on this concept, researchers have proposed cell-mimicking carriers. For instance, Cao *et al.* 128 designed DC membrane-coated nanoparticles capable of efficiently delivering mRNA to both the spleen and LNs following intramuscular injection. Furthermore, exosomes, which are natural carriers of functional small RNAs and proteins, have garnered significant interest in the field of drug delivery. These vesicles offer the potential to facilitate therapeutic delivery of miRNAs, siRNAs, mRNAs, lncRNAs, peptides, and synthetic drugs targeting the spleen.

Overall, emerging deformable nanocarriers with tunable size, aggregation, and stimuli-responsive properties have shown promise in enhancing drug delivery and therapeutic efficacy. However, intravenous administration of these carriers faces fatal challenges, such as retention and clearance by the livers, lungs, or kidneys, due to factors including particle size, surface charge, or modification layers 110, 129. In addition, prolonged or excessive spleen-targeted treatments may induce splenic complications, including malignancies, splenomegaly, and splenic dysfunction 130. Thus, further optimization and evaluation of these delivery systems are essential to maximize therapeutic efficacy while minimizing adverse effects.

## 5. Physical interventions on the spleen for disease immunotherapy

Physical medicine has emerged as a novel and promising strategy for direct immunomodulation or indirect neuro-immunoregulation of the spleen, offering an alternative to chemical interventions for immunotherapy targeting inflammatory and immune-related diseases. Currently, physical methods can be categorized into four main approaches: electrical stimulation, magnetic stimulation, photoirradiation, and ultrasonic stimulation.

### 5.1 Electrical stimulation

**Figure 8:**
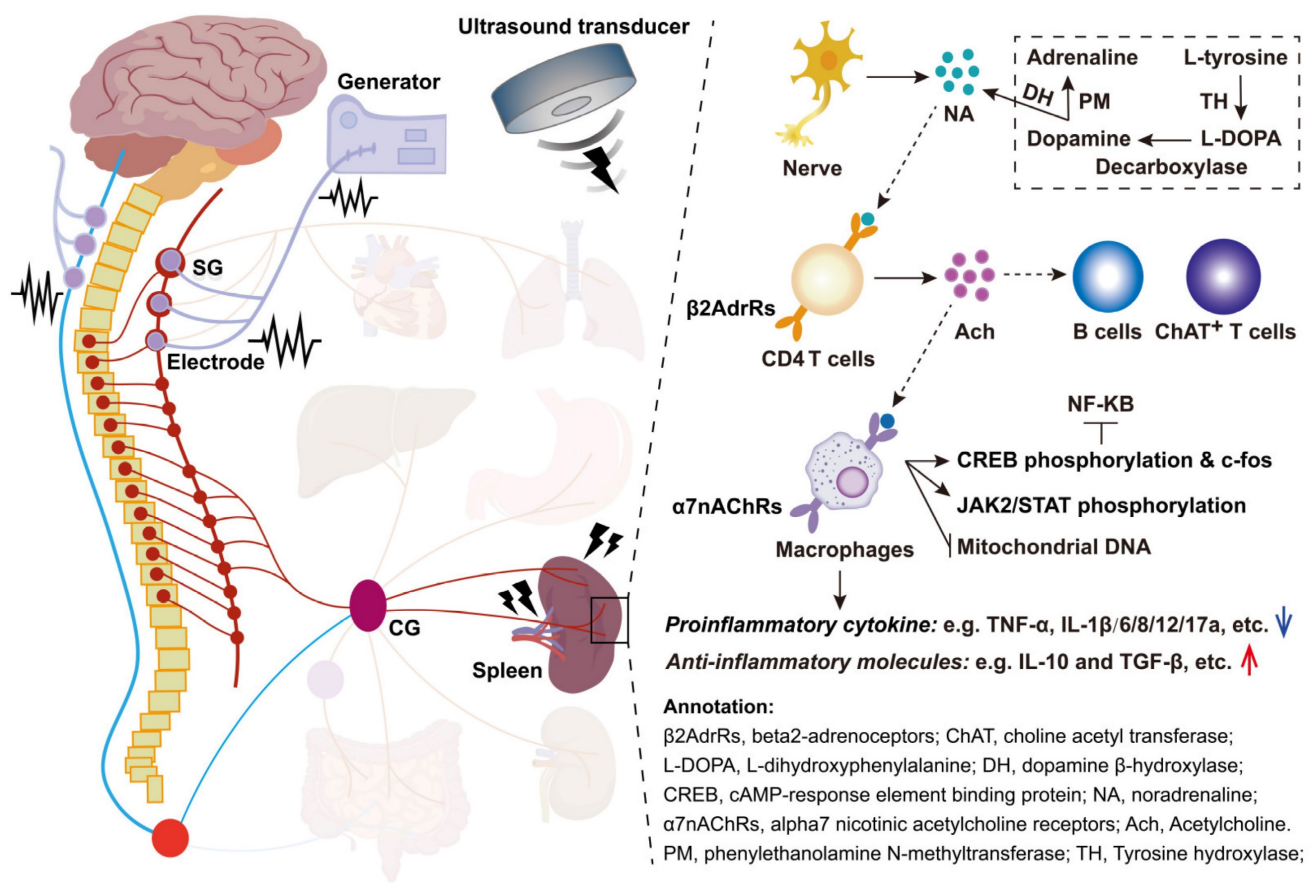
Activation of CAP by electrical cervical nerve stimulation and splenic ultrasound stimulation to combat inflammation. During this process, the sympathetic splenic nerves release NE, which binds to β2AdrRs on choline acetyltransferase (ChAT)-expressing CD4^+^ T cells. These T cells subsequently secrete Ach, which activates α7nAChRs on Mφs. The α7nAChR signaling pathway inhibits the release of pro-inflammatory cytokines, thereby reducing inflammation.

Rosas-Ballina *et al.* 86, 131 demonstrated that the CAP regulates TNF production in discrete Mφs via a dual-neuron circuit: a preganglionic neuron originating in the DMN of the vagus nerve and a postganglionic neuron from the celiac-superior mesenteric plexus, which projects into the splenic nerve. Specifically, they found that electrical vagus nerve stimulation (eVNS) during endotoxemia attenuates TNF production by splenic Mφs in the RP and MZ. Furthermore, their findings revealed that eVNS induces the release of Ach in the celiac mesenteric ganglia, which binds to the α7nAChR of the splenic nerve, leading to the release of NE in the spleen. NE subsequently binds to β2AdrR on splenic T lymphocytes, which release Ach that acts on α7nAChR in splenic Mφs to inhibit TNF-α release (Figure 8). Additional studies have corroborated the spleen's pivotal role in CAP-mediated immunomodulation via eVNS. Xue *et al.* 132 reported that eVNS provided no protective effect against septic shock in rats after splenectomy or celiac branch vagotomy. Similarly, Ji *et al.* 133 showed that cholinergic activation induced by the acetylcholinesterase inhibitor galantamine or a muscarinic Ach receptor agonist alleviated colitis in mice. However, this effect was abolished following vagotomy, splenic neurectomy, or splenectomy. Taken together, these findings underscore the critical mechanism of eVNS activating splenic CAP to modulate immunity and suppress inflammatory diseases. Herein, we summarized typical studies exploring the therapeutic potential of electrical stimulation of the vagus or sympathetic/splenic nerves to enhance splenic immunocompetence for various diseases (Table 3). Notably, eVNS has been shown to improve clinical outcomes in conditions such as polyneuropathies (NCT04053127), heart failure (NCT03425422), and traumatic brain injury (NCT02974959). Currently, eVNS methods include transcutaneous auricular vagus nerve stimulation and transcutaneous cervical vagus nerve stimulation, which modulate neural-immune-inflammatory responses in peripheral organs. These approaches have been comprehensively reviewed by Bonaz *et al.* 134.

Although numerous studies have established that the primary mechanism by which eVNS regulates splenic immunotherapy for various diseases involves CAP activation, this may not be the sole principle. For example, neural signals transmitted via the afferent vagus nerve can mitigate exacerbated “non-resolving inflammation” by acting on DCs and lymphocytes, as well as by suppressing NP accumulation 135, 136. In addition, Straub *et al.* 137 demonstrated that sympathetic neurotransmitters released in response to electrical stimulation can stimulate splenic T cells to secrete IFN-γ and the chemokine CXCL1 in type II collagen-induced arthritis. Similarly, MZBs responding to Streptococcus in the bloodstream were observed to migrate along splenic nerves to the RP venous sinuses, where they differentiated into antibody-secreting cells during eVNS 138. Guyot *et al.* 84 identified three nerve branches projecting to the spleen in mice, and two of these branches are associated with arteries and contain catecholaminergic fibers, while the third, located at the spleen's apex, contains both catecholaminergic and cholinergic fibers. Electrical stimulation of the apical nerve was found to inhibit inflammation independently of lymphocytes, instead relying on myeloid cells expressing adrenergic and nicotinic receptors. Moreover, the specific entry of calcium ions is an essential prerequisite for the release of NE during electrical stimulation, particularly with an increase in amplitude and frequency enhancing sympathetic nerve activity 139. Electrical stimulation of the splenic nerve also releases NE along with dopamine-β-hydroxylase, mediating neuro-immune interactions 140. Therefore, the immunomodulatory effects of eVNS may not be limited to Mφs but could also involve other splenic immune cells. Additionally, eVNS may stimulate the release of neurotransmitters such as dopamine, GABA, and 5-HT, rather than solely NE, to mediate neuro-immune interactions 141.

Numerous studies have demonstrated the potential of electrical stimulation to directly modulate the immune function of splenic lymphocytes. For instance, Piruzyan *et al.* 142 found that direct current suppressed the overproduction of pro-inflammatory cytokines induced by phorbol myristate acetate/ionomycin in Jurkat T cells and primary splenocytes. This intervention also improved liver damage and reduced spleen enlargement in a mouse model of concanavalin A-induced acute hepatitis. Additionally, Dong *et al.* 143 revealed that electrical stimulation activated the NF-κB pathway by stimulating ERK1/2 pathway, which promoted splenic B lymphocytes from the G0/G1 phase into the G2/S phase, indicating significant promotion of B lymphocyte proliferation in the spleen. Similarly, a recent study by Lee *et al.* 144 confirmed that the electrical stimulation enhanced the cytotoxic activity of NK cells by increasing calcium influx, thereby suppressing the proliferation of breast cancer MCF-7 cells. Furthermore, numerous studies have demonstrated the immunomodulatory effects of electrical stimulation on various other immune cells, further highlighting its therapeutic potential 145.

It is important to note that splenic neuro-immune modulation via electrical stimulation exhibits significant dose dependence. However, as shown in Table 3, there are no standardized electrical parameters applied for nerve stimulation to regulate splenic immunotherapy for diseases, leading to variable efficacy in both animal and clinical trials. Moreover, these inconsistencies are often accompanied by adverse effects. For example, Yamamoto *et al.* 146 reported significant seizure reduction with eVNS but also noted side effects such as hoarseness, throat discomfort, cough, paresthesia, and headache. Another challenge is that electrical currents delivered through microscopic electrodes can diffuse into adjacent areas, potentially affecting non-targeted structures and causing unintended side effects 147. Additionally, the electrical stimulation method requires direct physical contact between the electrode and tissue, which is combined with challenges in controlling the electrical current within biological tissues, often limiting the precision and efficacy of the intervention. Conventional methods are further hindered by fundamental limitations such as low spatial precision due to current dissipation in tissue and electrical artifacts that interfere with measurements 148. Notably, since cervical nerves innervate nearly all internal organs, electrical stimulation of these nerves may cause undesired biological effects in non-target organs. Taken together, these limitations underscore the need for further research to optimize electrical stimulation systems, particularly to develop precise and targeted stimulation of the splenic nerve or immune system with well-defined parameters.

Electroacupuncture (EA) shares similarities with electrical nerve stimulation (ENS), but the two modalities differ in key aspects that ENS typically stimulates a larger area and employs pulse widths that do not exceed 0.3 milliseconds, whereas EA often requires pulse widths of ≥ 0.3 milliseconds 149. Several studies have demonstrated the feasibility of EA in modulating splenic immunity for disease treatment. For instance, Wang *et al.* 150 showed that transcutaneous EA improved symptoms of ulcerative colitis, such as bloody or viscous stools and colonic inflammation, likely by regulating the balance between splenic Treg and Th17 lymphocytes in rats. Comparative studies indicate that EA outperforms ENS in long-term follow-up outcomes and quality of life improvement. However, ENS offers a noninvasive alternative and may be preferable for patients who are apprehensive about acupuncture 151.

### 5.2 Magnetic stimulation

Humankind has been exploring the application of magnetism for treating illnesses for thousands of years. Recent studies demonstrate that magnetic stimulation can directly modulate splenic immunocompetence. Fesenko *et al.* 157 and other colleagues 158 showed that exposure to electromagnetic waves significantly enhanced the cytotoxic activity of NK cells in the murine spleen. Similarly, Ogaĭ, *et al.* 159 reported that a single total-body exposure to electromagnetic centimeter waves (8.15-18 GHz, 1 mW/cm^2^, 5 hours) stimulated the proliferation of mouse splenic T and B lymphocytes.

This effect was also observed *in vivo* in rats exposed for 5 hours to millimeter waves (42.2 GHz, amplitude modulation 10 Hz, 1 mW/cm^2^). Murabayashi *et al.* 160 employed time-varying magnetic fields for cellular immunomodulation, finding significant activation of murine spleen lymphocytes, particularly during exposures of up to 40 minutes, which was likely mediated by calcium influx. Additionally, magnetic stimulation of the spleen holds promise for promoting the secretion of immunoregulatory factors, cell migration, and phagocytotic behavior. For example, TNF-α production in mouse spleens was significantly enhanced under stimulation conditions of pulse width = 238 ms, peak magnetic field = 0.25 T (at the center of the coil), frequency = 25 pulses/s, 1000 pulses/day, and magnetically induced eddy currents of 0.79-1.54 A/m^2^ 161. These findings are primarily based on the direct effects of magnetic stimulation on splenic immune cells. As summarized by Lei *et al.* 162, both innate and adaptive immune cells become more activated and initiate immune responses against diseases when subjected to various types of magnetic stimulation.

It is worth noting that numerous studies have demonstrated that magnetic stimulation can also activate the nervous system to treat psychiatric disorders and modulate host immunity against inflammatory disease, including epileptic seizures, atrial fibrillation, neuropathic and non-neuropathic pain, Parkinson's disease, stroke, and Alzheimer's disease 163, 164. However, few studies have directly explored the regulation of splenic immunity through magnetic stimulation of peripheral nerves. The vagus nerve, a mixed nerve comprising both afferent and efferent fibers, poses a rare but significant risk during magnetic stimulation, potentially leading to adverse events such as bradyarrhythmia and asystole 165. Moreover, the biophysical effects of magnetic stimulation on living cells vary considerably depending on magnetic field parameters, including homogeneity, intensity, and exposure duration 162. Therefore, further precise and large-scale investigations, encompassing both *in vitro* and *in vivo* studies across different types of magnetic fields, are essential before advancing toward clinical applications.

Magnetic stimulation acts on cells through multiple mechanisms, including hyperthermia responses, cytoskeletal remodeling, and ion channel gating alterations. Hyperthermia can induce the expression of heat shock proteins (HSPs) and immunologically significant molecules such as non-classical MHC antigens and IκB-α, while repressing the expression of pro-inflammatory cytokines such as IL-6, IL-1β, and TNF-α 166. These heat shock responses are transcriptionally regulated by a family of trans-activators, heat shock factors, which homotrimerize, translocate to the nucleus, and bind to heat shock elements in the promoter regions of HSP genes to activate transcription. Numerous studies have demonstrated the immunoregulatory effects of hyperthermia (39-41°C, similar to fever-range temperatures) on immune cells, involving enhanced T lymphocyte proliferation in response to IL-1/2 stimulus, increased IFN-γ production and cytotoxicity by effector CD8^+^ T cells against tumors, promotion of DC maturation, and upregulation of iNOS expression in Mφs 167, 168. Additionally, magnetic fields influence charge (spin) transfer in radical pair reactions by altering spin-state levels, triggering a cascade of biological responses. Paramagnetic free radicals and molecules, such as O3, NO, NO_2_, and FeCl_3_, are redistributed under the influence of the Lorentz force and magnetic gradient force, as understood from electrochemical principles 169. This redistribution can rearrange cellular components, including the actin cytoskeleton, Golgi complex, and the cation channel receptor TRPM2. For example, Wosik *et al.* 170 demonstrated that magnetic stimulation causes Mφs to cluster TRPM2 channels, disrupting Ca^2+^ homeostasis, which in turn alters ion current-dependent actin polymerization, leading to elongated cell phenotypes. Furthermore, Liu *et al.* 171 showed that magnetic stimulation redistributes vinculin within the cytoplasm and nucleus, while Zhang *et al.* 172 reported changes in the orientation and morphology of mitotic spindles in human cells, suggesting that magnetic torque affects both microtubules and chromosomes. Data regarding biological effects on voltage-gated ion channels also highlight the cellular impacts of magnetic fields. Panagopoulos *et al.* 173 reviewed evidence that magnetic stimulation alters intracellular concentrations of Ca^2+^, K^+^, Na^+^, and anions, which modulate immune cell biofunctionabilities such as proliferation, differentiation, reactive oxygen species (ROS) regulation, and apoptosis. Furthermore, magnetic stimulation has been proposed to influence iron absorption and the expression of iron storage-related proteins, including transferrin receptor-1 and ferritin, which play critical roles in Mφs-mediated immune homeostasis 174.

### 5.3 Photoirradiation

Photoirradiation-triggered neuronal activation represents a promising strategy for modulating neuroimmune interactions and alleviating inflammatory diseases 175. The mechanisms by which light activates nerves primarily involve direct actions, such as altering cell membrane potential, switching ion channels, stimulating neurotransmitter secretion, and regulating the expression of photosensitive molecules. Recent studies highlight the anti-inflammatory potential of optical nerve stimulation to modulate the splenic immunity. Tanaka *et al.* 76 demonstrated that laser irradiation of the vagus nerve significantly modulates immune responses in the spleen, mitigating acute kidney injury via activation of the C1 neuron-sympathetic nervous system-splenic nerve-spleen-kidney axis. Similarly, Demas *et al.* 176 confirmed that NE remarkably mediates photoperiodic lymphocyte proliferation via specific βAdrRs on splenic tissue.

In addition to neural effects, photoirradiation has been shown to directly enhance cellular biological activities, such as boosting mitochondrial ATP synthesis, inducing transient ROS production, regulating specific gene expression, improving cell survival, promoting proliferation and migration, and modulating intracellular Ca^2+^/Na^+^ levels 177. Fernandes *et al.* 178 and others 179 reported that laser irradiation reduces the expression of TNF-α, COX-2, iNOS, CCL3, and CXCL2, while increasing NO release in Mφs. Additionally, substantial evidence suggests that photobiomodulation with laser >660 nm wavelengths has also been associated with upregulated mitochondrial membrane potential, enhanced electron transport, greater oxygen consumption, increased synthesis of NADH and NADPH oxidase, and improved calcium dynamics, which can alter cell proliferation, translation and activation across diverse cell types. For instance, Xiong *et al.* 180 found that green monochromatic light enhances splenic T and B lymphocyte proliferation via melatonin pathways involving Mel1b, Mel1c, and RORα/RORγ receptors in chickens. Similarly, Boonstra *et al.* 181 reported that UVB irradiation impairs Th1-mediated immune responses *in vivo* by reducing systemic IL-12p70 levels and enhancing APCs to secrete prostaglandin E_2_, IL-1, IL-6 and TNF-α within the spleen.

However, photoirradiation outcomes may vary based on disease model personalization, radiation parameters, or individual responses. For instance, Xie *et al.* 182 found that red light stimulation of the subdiaphragmatic vagus nerve in aged septic mice induced splenomegaly, disrupted gut microbiota, and exacerbated learning impairments and anxiety-like behaviors. These findings highlight the need for precise optimization of wavelength, dosage, and exposure protocols to maximize therapeutic benefits while minimizing adverse effects.

### 5.4 Ultrasound stimulation

The above findings indicate the significant efficacy and medical value of physical methods for splenic neuro- and immunomodulation in treating various diseases. However, these methods are not without limitations, as they may induce adverse reactions during application. More critically, their clinical translation and widespread use are constrained by inherent physical limitations. For instance, photoirradiation penetrates to a depth of only 3-5 cm, with approximately 6.6% of 670 nm laser photons and 11.3% of 830 nm laser photons reaching the target tissue 183-185. Thus, there is a pressing need for a non-invasive, targeted, and precisely controlled physical method capable of extensively activating splenic immunocompetence without toxic side effects. Notably, ultrasound has emerged as a promising modality due to its advantages, including deep tissue penetration, non-invasive application, precise targeting, controllability, and real-time monitoring enabled by unique ultrasound echo signals. These characteristics, combined with significant biophysical effects, make ultrasound an ideal tool for neuro- and immunomodulation to regulate splenic immunocompetence against diseases.

**Figure 9:**
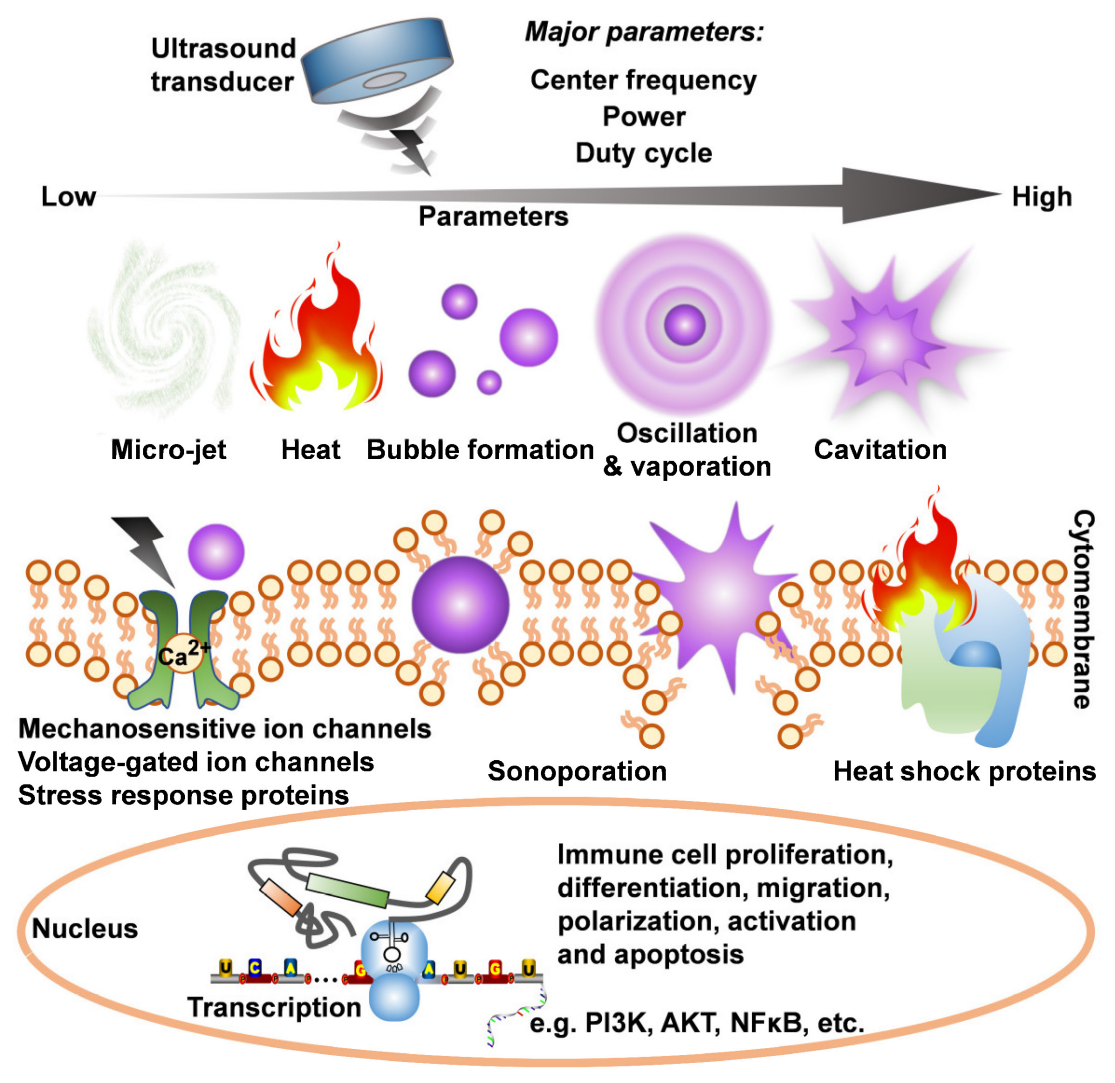
Schematic diagram of the physical mechanism of ultrasound and its biophysical effects.

Ultrasound exhibits a dual mechanism in immunoregulation: (1) direct stimulation of splenic lymphocytes (Figure 9), and (2) modulation of neuro-immune networks to indirectly enhance splenic immunocompetence (Figure 8). Since Gigliotti *et al.* 186 first demonstrated in 2013 that ultrasound can mitigate renal ischemia-reperfusion injury by stimulating the splenic CAP, research on “splenic ultrasound stimulation (SUS)” for disease immunotherapy has garnered increasing global attention over the past decade. As summarized in Table 4, SUS holds significant potential for treating a range of conditions, including rheumatoid arthritis, colitis, pneumonia, hyperglycemia, and other inflammatory diseases. Most studies indicate that SUS significantly activates CAP, influencing splenic CD4^+^ T cells and Mφs to suppress the secretion of inflammatory cytokines, thereby countering disease progression. Irrationally, the majority of research has focused on the SUS modulation of inflammatory mediators, such as TNF-α, IL-6, and IL-10, as well as on CAP validation, but performed comparatively less exploration of the dynamic transitions occurring in splenocytes during SUS (Figure 8), highlighting a gap that warrants further investigation.

Several studies have reported significant alterations in the proportions of T cells and B cells, but not Mφs, in the spleen following SUS 187, 188. Generally, ultrasound irradiation directly affects cells by modulating the expression of key genes and signaling pathways, interfering with cytokine secretion, and facilitating ion influx to regulate cellular biological processes 189, 190. Beyond its splenic neuro-immunomodulatory mechanisms (e.g., CAP), SUS likely promotes immune cell proliferation, activation, migration, infiltration, and phagocytosis directly through ultrasonic mechanical forces, microjets, vaporization, and cavitation effects within the splenic microenvironment (Figure 9). Notably, Xia *et al.* 191 demonstrated that SUS directly activates splenic CD4^+^ and CD8^+^ T cells in 4T1 tumor-bearing mice, enhances tumor-infiltrating lymphocyte accumulation, and modulates cytokines and chemokines in the tumor microenvironment, thereby suppressing tumor growth. This finding suggests a promising role for SUS in manipulating splenic immunity to restore immunocompetence and reverse the immunosuppressive state in tumors, potentially improving immunotherapy outcomes. Additionally, numerous studies have confirmed that ultrasound facilitates calcium influx, which alters intracellular calcium-dependent signaling pathways to elicit specific biological effects 192, including those involved in neural and immune responses 78.

SUS effects demonstrate significant dose dependence and tolerance (Table 4). For example, Liu *et al.* 193 optimized an ultrasonic parameter of 0.35 MPa with a 1-second on/5-second off duty cycle, which significantly alleviated autoimmune myocarditis and regulated the proportions and functions of Tregs and Mφs through CAP activation. Similarly, Cotero *et al.* 194 found that non-invasive SUS at 0.83 MPa effectively reduced arthritis severity and attenuated cytokine responses to endotoxins by modulating CAP via CD4^+^ T cells and Mφs. Despite these promising findings, the ultrasonic parameters used for spleen stimulation remain highly variable across studies. Many studies rely on parameters reported in previous research without independent screening or optimization. For instance, Hu *et al.* 188 and Morton *et al.* 195 adopted the 0.35 MPa and 1-second on/5-second off duty cycle parameters reported by Zachs *et al.* 187 for splenic immunomodulation to treat inflammatory diseases. However, practical applications reveal significant differences in ultrasonic focal regions depending on the ultrasound platform. Current parameters for SUS are largely unvalidated, with fewer than 10 studies providing systematic evidence (as summarized in Table 4), which severely limits clinical translational progress. Two clinical trials investigating SUS-mediated immunotherapy for inflammatory diseases (NCT03690466 and NCT03548116) are currently registered on ClinicalTrials.gov, although their results have not yet been officially published. We look forward to their positive outcomes that would pave the way for widespread clinical adoption of SUS to alleviate patient suffering.

## 6. Challenges and perspectives

As demonstrated above, the functional regulation of the spleen holds significant potential for clinical applications. In this study, we have systematically compiled a curated selection of representative clinical trials, as presented in Table 5, which encapsulates spleen-targeted therapeutic strategies for disease diagnosis and treatment. These trials encompass diverse modalities, including physical stimulation, chemotherapy, small molecule inhibitors, traditional Chinese medicine, and other immunomodulatory interventions. The target indications span hematologic disorders, malignancies, and inflammatory diseases, highlighting the spleen's emerging role in systemic disease modulation. Although several studies remain in early-phase development, others have advanced to late-stage clinical evaluation, highlighting the translational potential of spleen-targeted therapies.

Despite significant advances in spleen-targeted immunomodulation, several challenges must be addressed to facilitate clinical translation 200. A primary obstacle lies in the intricate neuroimmune interactions within the spleen, which remain incompletely understood. The heterogeneity of splenic immune cell populations and their dynamic responses to local and systemic signals introduce variability in therapeutic outcomes. Current physical (e.g., electrical and ultrasound stimulation), genetic, and pharmacological strategies for splenic modulation require further optimization to achieve precise, reproducible, and patient-specific effects. Advances in high-resolution imaging, optogenetics, and bioelectronic medicine may improve the spatial and temporal precision of spleen-targeted interventions.

Regulatory and translational hurdles also pose significant challenges. The complex nature of spleen-targeted therapies necessitates rigorous validation to meet safety and efficacy standards. Biocompatibility, long-term immune consequences, and the potential for off-target effects must be thoroughly evaluated. Furthermore, interindividual variability in splenic architecture and immune composition may influence treatment responsiveness, highlighting the need for personalized therapeutic strategies. The integration of nanomedicine-based delivery platforms, artificial intelligence-driven predictive modeling, and multi-omics approaches could enhance patient stratification and treatment personalization.

Future research should focus on refining therapeutic parameters, developing non-invasive stimulation techniques, and improving drug delivery systems to maximize efficacy while minimizing adverse effects. A multidisciplinary effort combining immunology, bioengineering, and computational modeling will be essential to accelerate the translation of spleen-targeted immunotherapies into clinical practice.

## 7. Conclusions

The spleen serves as a central hub for immune regulation, with diverse immunocytes that orchestrate host defense, inflammation, and tissue homeostasis. Its extensive neuroimmune interactions, particularly those mediated by sympathetic innervation, provide a unique opportunity for therapeutic intervention. Although early research explored splenectomy as a disease-modifying strategy, subsequent studies have highlighted the spleen's indispensable role in maintaining immune balance. Recent advancements in physical, genetic, and pharmacological modulation of splenic immunity have demonstrated significant potential in treating neurological, inflammatory, cardiovascular, autoimmune, and oncological diseases. Notably, bioelectronic medicine, including splenic nerve stimulation, represents a promising frontier for precise immune regulation. However, overcoming challenges related to targeted delivery, interpatient variability, and regulatory approval is crucial for clinical translation. By utilizing emerging technologies such as nanomedicine, bioinformatics, and artificial intelligence, future research can optimize spleen-targeted therapeutic strategies, paving the way for next-generation immunomodulatory interventions.

## Figures and Tables

**Table 1:** Genetic and chemical immunomodulation of the spleen for disease treatment.

No.	Intervention type	Mechanism of action	Disease target	Reported efficacy	Potential side effects	Ref.
1	RNA vaccine lipoplexes (HA-LPX, gp70-LPX, OVA-LPX)	Induces proliferation of antigen-specific CD8+ and CD4+ T cells in the spleen	Cancer immunotherapy	30-60% CD8+ T cell activation	Potential for off-target immune responses	90
2	γ-PGA-coated pUb-M DNA vaccine	Selective transgene expression in the splenic marginal zone (MZ) to inhibit tumor growth	Melanoma	Significant tumor growth inhibition	Requires optimized delivery for clinical use	91
3	CRISPR gene editing	Targeted gene modification in splenic lymphocytes to enhance immunity	Autoimmune and genetic disorders	Preclinical success	Off-target effects, immunogenicity	92
4	siRNA silencing of MyD88	Suppresses inflammatory cytokine production in splenic cells	Inflammatory diseases	Reduced IL-1β, TNF-α, IL-8, and NF-κB levels	Transient effects, potential immune suppression	93
5	CCR2-silencing siRNA in lipid nanoparticles	Reduces monocyte-driven inflammation	Cardiovascular disease, diabetes, cancer	Reduced infarct size, prolonged normoglycemia, decreased tumor growth	Dose-dependent immune modulation	94
6	JAK inhibitors (e.g., Tofacitinib, Ruxolitinib)	Inhibits JAK-STAT signaling to modulate splenic immune function	Autoimmune diseases (rheumatoid arthritis, psoriasis)	40-70% reduction in inflammation	Risk of infections, thrombosis	95, 96
7	S1P receptor modulators	Regulates immune cell migration from the spleen	Inflammatory disorders	Reduced inflammation	Potential cardiovascular risks	95, 96
8	Adrenergic receptor antagonists (Carvedilol, Propranolol)	Blocks brain-spleen-brain cycle to reduce stroke-induced immune suppression	Stroke recovery	Reduced spleen atrophy and infarction size	Hypotension, fatigue	97
9	Spleen tyrosine kinase (Syk) inhibitors (Fostamatinib, HMPL-523, Cevidoplenib)	Modulates immune signaling in splenic immune cells	Autoimmune hemolytic anemia, ITP, vasculitis, COVID-19 inflammation	Effective in multiple immune-related diseases	Risk of infections, liver toxicity	98
10	17β-Estradiol	Enhances splenic B cell responses to TLR9 agonists	Lupus	Improved B cell function	Hormonal side effects	99
11	Pioglitazone (PPAR-γ agonist)	Increases Treg populations and reduces inflammatory T cells in the spleen	Schistosoma-induced pathology	Decreased inflammation	Metabolic side effects	100
12	Herbal compounds (Licochalcone A, Artemisinin, Eucommia ulmoides extracts)	Stimulates splenic T and B cell proliferation	Immune enhancement	Preclinical immune activation	Varies by compound	101, 102
13	Immunostimulants targeting TLR2, TLR4, TLR5, TLR7, TLR8, TLR9	Enhances splenic T cell responses	Infectious diseases, cancer	Improved immune response	Risk of overactivation	103, 104
14	Protease inhibitors targeting splenic macrophages	Suppresses excessive immune activation	Autoimmune diseases	Reduction in systemic inflammation	Immune suppression	105, 106
15	αCD147 treatment	Eliminates inflammatory spleen-derived monocytes	Stroke recovery	Reduced immune-mediated brain injury	Potential off-target effects	97
16	Recombinant IL-33 therapy	Regulates splenic T cell responses, promoting Treg function	Autoimmune diseases	Increased Treg activity	Unknown long-term effects	97
17	RTL551/1000 therapy	Prevents splenic atrophy and immune cell mobilization	Neuroinflammatory diseases	Improved immune regulation	Under investigation	97
18	Anti-GITR and CD28 superagonist combination	Enhances IL-10-producing T cell populations in the spleen	Inflammatory bowel disease	Reduced inflammation in DSS models	Risk of immune overactivation	107
19	Agonistic OX40 monoclonal antibody	Expands effector CD8+ and CD4+ T cells for enhanced immune response	Vaccine enhancement	Increased protective immunity	Potential for hyperinflammation	108
20	Immune checkpoint inhibitors (e.g., Anti-PD-1, Anti-PD-L1, Anti-CTLA-4)	Restores T cell function by blocking inhibitory signals	Cancer immunotherapy	Improved tumor response rates	Autoimmune-like toxicities	109

**Table 2:** Comparison of emerging spleen-targeted nanomedicines for disease theranostics.

Nanomedicine type	Targeting mechanism	Therapeutic application	Advantages	Limitations
Lipid-based nanoparticles (e.g., liposomes, solid lipid nanoparticles)	Passive targeting via enhanced retention in the spleen; surface modification for active targeting (e.g., mannose-functionalization for APC uptake)	Cancer immunotherapy, infectious disease treatment, vaccine delivery	High biocompatibility, ability to encapsulate hydrophilic and hydrophobic drugs, controlled drug release	Potential instability, rapid clearance by the mononuclear phagocyte system (MPS)
Polymeric nanoparticles (e.g., PLGA, PEGylated nanoparticles)	Passive targeting via splenic filtration; active targeting using ligand-modified surfaces (e.g., anti-CD169, DEC-205 antibodies)	Autoimmune diseases, cancer immunotherapy, inflammation modulation	Biodegradable, tunable drug release kinetics, enhanced stability	Potential for off-target effects, variability in biodistribution
Inorganic nanoparticles (e.g., gold, silica, iron oxide)	Surface functionalization for active targeting; magnetic targeting (for iron oxide)	Theranostics (imaging + therapy), targeted drug delivery	High imaging contrast for diagnostics, potential for photothermal therapy	Potential toxicity, long-term accumulation concerns
Extracellular vesicle-based delivery (e.g., exosomes, synthetic vesicles)	Natural homing ability to the spleen, functionalization for enhanced targeting	RNA-based therapies, immunomodulation, regenerative medicine	High biocompatibility, endogenous origin reduces immune rejection, ability to cross biological barriers	Complex isolation and scalability, potential heterogeneity
Hydrogel-based nanomedicine	Localized controlled-release within the spleen, immune cell-mediated uptake	Cancer immunotherapy, sustained vaccine delivery	Prolonged retention, tunable mechanical properties, minimal systemic toxicity	Potential for slow degradation, manufacturing complexity

**Table 3:** Summary of representative studies on the application of eVNS in regulating splenic immune responses to inflammatory diseases.

No.	Disease models	Electrical stimulation methods and parameters	Therapeutic effects	Mechanisms	Ref.
1	Kidney ischemia reperfusion injury in mice	Implantable electrical stimulation (square wave; 50 μA intensity; frequency, 5 Hz; duration, 1 ms) was applied for 10 minutes.	Stimulation of vagal afferents or efferent nerves 24 hours before ischemia-reperfusion injury markedly attenuated acute kidney injury and reduced plasma TNF.	VNS-mediated attenuation of acute kidney injury and systemic inflammation depends on α7nAChR positive splenocytes.	87
2	Bleeding in hemophilia A mice	The nerve was mounted on bipolar platinum electrodes, which were used to apply constant voltage stimuli (1 V, 30 Hz, 2 ms pulse width) for five minutes.	VNS primes platelets and reduces bleeding in hemophilia A.	VNS targets Ach-producing T lymphocytes in spleen and α7nAChRs on platelets to increase calcium uptake and enhance alpha granule release.	152
3	Acute pancreatitis in mice	Implantable electrical stimulation, using a biphasic square waveform (cathodic first, 500 μs total pulse duration, 225 μs for each phase, and a 50 μs interphase delay), was applied at 10 Hz for a duration of 2 minutes.	VNS reduced plasma lipase and amylase activities, blunted the concentrations of TNF-α and protected against pancreas histologic damage in acute pancreatitis models.	VNS increased the percentages of α7nAChR+ Mφs in the pancreas and spleen. Adoptive transfer of VNS-treated α7nAChR splenocytes conferred protection against pancreatitis in recipient mice.	153
4	Angiotensin II infusion of mice	The implantable electrodes were applied to stimulate the vagus nerve every other minute within a 10-minute stimulation window, with a stimulation frequency of 5 Hz, and a stimulation pattern generated by a monophasic pulse of 0.3 mA.	Bioelectronic stimulation of the celiac vagus nerve evoked the noradrenergic splenic pathway to promote the release of a growth factor mediating neuroimmune crosstalk, placental growth factor (PlGF), and egress of CD8 effector T cells.	The splenic neuroimmune interface mediated by PlGF and necessary for transducing the neural signal into an effective immune response is dependent on α-adrenergic receptor signaling.	154
5	Sepsis in mice	The electrical stimulation parameters included: 1 V, 2 ms, 5 Hz for 20 minutes (10 minutes before LPS and 10 minutes after); 1 V, 2 ms, 5 Hz for 2 minutes (1 minute before LPS and 1 minute after); 1 V, 2 ms, 5 Hz for 30 seconds (5 minutes after LPS); and 1 V, 0.5 ms, 30 Hz for 30 seconds (5 minutes after LPS).	Transcutaneous VNS dose-dependently reduced systemic TNF levels, HMGB1 levels and improved survival in mice with polymicrobial sepsis.	Electrical VNS inhibited proinflammatory cytokine production and prevented shock during lethal systemic inflammation through the α7nAChR-dependent pathway to the spleen, known as the CAP.	155
6	Rheumatoid arthritis in mice	The implantable electrostimulation used rectangular, charge-balanced biphasic pulses with a pulse amplitude of 650 µA, a pulse width of 100 µs (for both positive and negative phases), and a frequency of 10 Hz, applied for 2 minutes (STIM).	Electrical stimulation of the splenic nerves inhibited inflammation independently of lymphocytes.	The inhibition of inflammation by splenic nerve electrical stimulation relied on signaling by both β2AdrRs and α7nAChRs in myeloid cells.	84
7	Middle cerebral artery occlusion (MCAO) in rats	A 30-second train of stimulation consisting of 0.5 ms square pulses (0.5 mA) delivered at 20 Hz was initiated 30 minutes after middle cerebral artery occlusion (MCAO). Stimulation was repeated every 5 minutes for a duration of 1 hour.	The ta-VNS treatment improved recovery of neurological function, reduced infarct volume, and induced angiogenesis, potentially through GDF11 mobilization, with effects mediated by ALK5.	The ta-VNS improved neurobehavioral recovery, upregulated cerebral GDF11 and downregulated splenic GDF11, indicating a brain-spleen communication during stroke.	156

**Table 4:** Summary of some typical literature applying SUS for various disease immunotherapies.

No.	Summary of experimental studies	Ultrasonic parameters	Therapeutic effects and mechanisms	Ref.
1	Acute colitis was induced by administering 2% Dextran Sulphate Sodium (DSS) in drinking water for 7 days, followed by therapeutic ultrasound applied to the abdominal area.	A 5 cm^2^ transducer (Mettler 740×; Anaheim, CA), operating at 1 MHz, with a 10% duty cycle and an intensity of 2 W/cm^2^, was applied for 7 minutes daily from day 4 to day 10.	Ultrasound improved DSS-induced colitis by stimulating the splenic nerve and activating the CAP, leading to a reduction in colonic F4/80^+^α7nAChR^+^ Mφ.	196
2	Rheumatoid arthritis was induced in 9-week-old DBA/1J mice by administering type II collagen emulsified with an equal volume of complete Freund's adjuvant. Ultrasound was applied to the spleen of collagen-injected animals on day 0.	A 1-MHz transducer operating at 350 kPa with bursts of 1 second on/5 second off (16.7% duty cycle) was applied for 2 minutes daily over a period of 10 consecutive days.	Spleen-targeted low-frequency pulsed focused ultrasound (FUS) effectively alleviated arthritis severity, eliciting a distinct response in T cell subsets, particularly CD8^+^ T cells, and myeloid cell subsets to ultrasound stimulation.	197
3	Pulmonary hypertension was induced in rats either by injecting Sugen 5416 (20 mg/kg SQ) followed by 21 or 35 days of hypoxia (Sugen/hypoxia model), or by injecting monocrotaline (60 mg/kg IP) (monocrotaline model). FUS was applied to the spleen in 12-minute daily sessions.	A 1.1 MHz focused transducer, with a 0.83 MPa (200 mVpp amplitude) pulsed sinusoidal waveform, a 0.5 ms pulse repetition period (150 cycles), was applied from day 11 to day 24.	FUS stimulation of the spleen improves hemodynamic, autonomic, laboratory, and pathological outcomes in two experimental pulmonary hypertension models. This effect is associated with the normalization of CD68^+^ and CD8^+^ T cell counts in the spleen, as well as the downregulation of several inflammatory genes and pathways in nonclassical and classical monocytes and Mφs in the lung.	198
4	Autoimmune myocarditis was induced using a cardiac-specific peptide, and low-intensity pulsed ultrasound was used to stimulate the splenic nerve.	A concave transducer (OLYMPUS V302) operating at 1 MHz and pressures of 0.1 MPa, 0.35 MPa, and 0.473 MPa, with 1-second on/5-second off bursts, was applied for either 6 or 12 minutes daily from day 0 to day 21, or from day 7 to day 21.	Splenic ultrasound reduced heart inflammation and improved cardiac remodeling by alleviating the immune response, as well as regulating the proportion and function of CD4^+^ Tregs and Mφ through the activation of CAP.	193
5	Pneumonia was induced by lung instillation of 10^5^ CFU of *Streptococcus pneumoniae*, and splenic ultrasound stimulation was administered at 4, 16, or 48 hours after bacterial inoculation.	A transducer (Sonic Concept H106) operating at 1.1 MHz, with a 200 mVpp amplitude, 150 burst cycles, and a burst period of 200 ms.	Non-invasive ultrasound stimulation targeted to the spleen demonstrated a time-dependent effect on CAP activation via modulating the cytokine response throughout the progression of infection.	199
6	Full thickness cutaneous excisional wounds were constructed in a rodent model of type II diabetes, and FUS pulses were applied externally to the spleen area for 3 minutes daily over a 15-day period.	A transducer (Sonic Concept H106) operating at 1.1 MHz with a 300 mVpp amplitude and a pulse repetition interval of 0.5 ms.	Non-invasive splenic pFUS accelerated wound closure in type II diabetes rodents by up to 4.5 days compared to sham controls via CAP modulation.	195
7	Acute inflammation and metabolic dysfunction were induced by LPS injection, and FUS was used for splenic nerve stimulation.	A transducer (Sonic Concept H106) operating at 1.1 MHz with a 0.27 duty cycle, 136.36 µs pulse length, 0.83 MPa peak positive pressure, delivering a single stimulus not exceeding a 1-minute pulse.	Ultrasound applied to the spleen reduced the cytokine response to endotoxin to levels similar to those achieved with implant-based vagus nerve stimulation via CAP modulation.	194
8	Inflammatory arthritis was induced by administering arthritogenic donor serum, and ultrasound irradiation of the spleen was performed one day prior to serum transfer.	A single element ultrasound transducer operating at 1 MHz with a 350 kPa pressure, 1-second on/5-second off pulse pattern, applied for 2 minutes per day over 7 consecutive days.	Ultrasound stimulation targeting the spleen significantly reduced disease severity in a mouse model of inflammatory arthritis, primarily due to the anti-inflammatory effects of splenic T and B cells.	187
9	RA was induced by type II collagen, and FUS irradiation of the spleen was performed one day prior to collagen injection.	A FUS concave transducer (Panametrics-NDT, Waltham, MA, USA) operating at 1 MHz with 350 kPa pressure, 1-second on/5-second off bursts, applied for 12 minutes per day.	Spleen-targeted low-frequency pulsed FUS effectively alleviated arthritis severity by regulating various cell subpopulations, particularly CD8^+^ T cell subsets.	188

**Table 5:** Clinical trials investigating spleen-targeted therapeutic strategies for disease theranostics.

Clinical trial ID	Therapeutic approach	Target disease	Phase/type	Status
NCT05363007	Radiation + nanoliposomal irinotecan	Pancreatic cancer	Phase II	Active
NCT05735834	Rituximab + zanubrutinib	Splenic marginal zone lymphoma	Phase III	Active
NCT05805358	Hyperpolarized 13C MRI cover spleen	Gynecological cancer	Phase II	Active, not recruiting
NCT06243159	JY231 + chemotherapy	Autoimmune diseases	Phase I	Active, not recruiting
NCT05980806	Selinexor monotherapy	Myelofibrosis and Moderate Thrombocytopenia	Phase II	Active
NCT03991780	Fostamatinib	Chronic active antibody mediated rejection in renal transplantation	Phase II	Active, not recruiting
NCT03165734	Pacritinib + physician's choice therapy (e.g., JAK2 inhibitor)	Thrombocythemia myelofibrosis	Phase III	Active
NCT01882933	Curative gastrectomy + hyperthermic intraperitoneal chemoperfusion	Gastric adenocarcinoma	Phase III	Active, not recruiting
NCT06418256	Spleno-pancreectomy, or a total or partial splenectomy	Malaria	Observational	Active
NCT06189066	Spleen ultrasound stimulation	COVID-19	Not applicable	Active, not recruiting
NCT06553053	Acupuncture	Crohn's disease	Not applicable	Active
NCT05003310	Electrical stimulation	Rheumatoid arthritis	Not applicable	Active
NCT04955899	Electrical stimulation of splenic neurovascular bundle	Rheumatoid arthritis	Not applicable	Active
NCT06074718	Pricking therapy + wheat grain moxibustion	Malignant tumors	Not applicable	Active
NCT05685108	Ultrasonic stimulation of splenic nerve in hilum	Autoimmune conditions	Not Applicable	Active

## Abbreviations

DCs: dendritic cells
NPs: neutrophils
MDSCs: myeloid-derived suppressor cells
Mφs: macrophages
RBC: red blood cell
HSPCs: hematopoietic stem and progenitor cells
RP: red pulp
WP: white pulp
MZ: marginal zone
PFZ: perifollicular zone
LNs: lymph nodes
CA: central artery
TCZ: T cell zone
BCZ: B cell zone
GCs: germinal centers
SLO: secondary lymphoid organ
BCs: bridging channels
BM: bone marrow
LPS: lipopolysaccharide
HPA: hypothalamic-pituitary-adrenal
SAM: sympatho-adrenal medullary
Ach: acetylcholine
NE: norepinephrine
β(2)AdrRs: β(2)-adrenergic receptors
α7nAChR: α7 nicotinic acetylcholine receptor
CAP: cholinergic anti-inflammatory pathway
ChAT: choline acetyltransferase
ANS: autonomic nervous system
CNS: central nervous system
SNS: sympathetic nervous systems
PaSNS: parasympathetic nervous systems
CG: celiac ganglion
eVNS: electrical vagus nerve stimulation
ENS: electrical nerve stimulation
EA: electroacupuncture
SUS: splenic ultrasound stimulation
